# Effectiveness and safety of intravitreal faricimab for macular oedema secondary to retinal vein occlusion: a systematic review and meta-analysis

**DOI:** 10.1186/s40942-026-00899-y

**Published:** 2026-07-09

**Authors:** Ali Alseneid, Shivam Bhindi, Mohammad Saleki, Diya Baker

**Affiliations:** 1 William Harvey Hospital, Ophthalmology, East Kent Hospitals University NHS Foundation Trust, Kennington Road, Ashford, Kent, TN24 0LZ England, UK; 2 King’s College London School of Medical Education, London, England, UK; 3 East Lancashire Hospitals NHS Trust, Ophthalmology, Burnley, England, UK

**Keywords:** Faricimab, Retinal vein occlusion, Macular oedema, Branch retinal vein occlusion, Central retinal vein occlusion, Anti-VEGF, Treat-and-extend, Real-world evidence, Systematic review, Meta-analysis

## Abstract

**Background:**

Faricimab is a bispecific monoclonal antibody targeting both vascular endothelial growth factor A and angiopoietin-2, approved for macular oedema secondary to retinal vein occlusion in 2023. The phase III BALATON and COMINO trials demonstrated non-inferiority to aflibercept at week 24, and several real-world cohorts have subsequently emerged. The aim of this systematic review and meta-analysis was to estimate the pooled effect of faricimab on visual acuity, treatment burden, anatomical outcomes, and safety in this indication.

**Methods:**

PubMed, EMBASE, and the Cochrane Central Register of Controlled Trials were systematically searched from inception to 1 May 2026 for studies reporting outcomes following intravitreal faricimab. Risk of bias was assessed in duplicate using the Cochrane Risk of Bias 2 tool for the randomised evidence and the Risk Of Bias In Non-randomised Studies of Interventions tool for the observational evidence, with single-arm cohorts evaluated as pre-post comparisons. The primary visual acuity outcome was pooled using random-effects meta-analysis with Hartung-Knapp-Sidik-Jonkman adjustment, stratified by treatment status, while outcomes precluded from pooling by methodological heterogeneity were synthesised narratively. Certainty of evidence was assessed using the Grading of Recommendations, Assessment, Development and Evaluation framework.

**Results:**

Ten studies comprising 1,620 eyes met eligibility criteria, including the BALATON and COMINO randomised controlled trials and nine non-randomised cohorts. The pooled mean change in best-corrected visual acuity at approximately 6 months was + 16.89 Early Treatment Diabetic Retinopathy Study letters (95% confidence interval 16.05 to 17.72) in treatment-naïve eyes and + 8.73 letters (95% confidence interval 4.75 to 12.71) in refractory switch cohorts, with negligible between-study heterogeneity in both analyses. Narrative synthesis was consistent with treatment interval extension following initiation of or switch to faricimab, statistically significant reductions in central retinal thickness across studies, and a short-term safety profile without identified retinal vasculitis events.

**Conclusion:**

The synthesis is consistent with intravitreal faricimab use being associated with visual and anatomical improvement in macular oedema secondary to retinal vein occlusion, with the treatment-naïve pooled estimate primarily reflecting registration trial data and the switch cohort estimate characterising refractory phenotypes.

**Supplementary Information:**

The online version contains supplementary material available at https://doi.org/10.1186/s40942-026-00899-y.

## Introduction

Retinal vein occlusion (RVO) is the second most common retinal vascular disease after diabetic retinopathy and a leading cause of vision loss in adults of working age, with an estimated global prevalence of approximately 28 million people [1]. Branch retinal vein occlusion (BRVO) accounts for the majority of cases globally, while central retinal vein occlusion (CRVO) and hemiretinal vein occlusion (HRVO) represent less common but typically more severe disease phenotypes [2]. Macular oedema is the principal driver of visual impairment in this population, occurring in up to 60% of eyes with BRVO and 75% of eyes with CRVO within the first 12 months following the index event [3]. The pathophysiology of macular oedema secondary to RVO is multifactorial: venous obstruction leads to capillary endothelial dysfunction, retinal ischaemia, and elevated intravitreal concentrations of vascular endothelial growth factor (VEGF), angiopoietin-2 (Ang-2), and inflammatory cytokines, which together promote vascular leakage and disrupt the blood-retinal barrier [4, 5].

Intravitreal anti-VEGF therapy is the current first-line treatment for macular oedema secondary to RVO, with ranibizumab and aflibercept established as effective agents through the BRAVO, CRUISE, BRIGHTER, HORIZON, VIBRANT, and COPERNICUS pivotal trials [6–9]. While these agents reliably improve visual acuity and reduce central retinal thickness, real-world evidence has consistently demonstrated a gap between trial efficacy and clinical effectiveness, with patients in routine practice receiving fewer injections and achieving smaller visual gains than those in registration studies [10, 11]. Treatment burden is a particular limitation: the requirement for frequent intravitreal injections, often monthly during the loading phase, places a substantial demand on patients, caregivers, and healthcare systems, and has been associated with reduced adherence, undertreatment, and suboptimal long-term visual outcomes [12, 13]. A subset of eyes additionally develop persistent or recurrent macular oedema despite continued anti-VEGF therapy, with structural and functional ceiling effects emerging in chronically treated patients [14]. These limitations have motivated the development of therapies targeting additional pathogenic pathways beyond VEGF alone.

Faricimab (Vabysmo, Roche/Genentech) is the first humanised bispecific monoclonal antibody designed for intraocular use, independently binding and neutralising both VEGF-A and Ang-2 [15]. Preclinical studies have demonstrated that simultaneous inhibition of both pathways promotes more durable vascular stabilisation and reduces inflammatory leakage to a greater extent than VEGF inhibition alone, providing the mechanistic rationale for improved treatment durability in retinal vascular diseases [16, 17]. The phase III BALATON and COMINO trials, conducted in patients with macular oedema secondary to BRVO and to CRVO or HRVO respectively, demonstrated non-inferiority of faricimab 6.0 mg compared with aflibercept 2.0 mg for visual acuity gain at 24 weeks of monthly dosing, with adjusted mean BCVA gains of approximately 17 ETDRS letters across both arms in both trials [18]. In the subsequent week 24 to 72 single-arm extension phase reported by Danzig et al., eyes transitioned to a modified treat-and-extend regimen, with the majority of patients in BALATON achieving treatment intervals of 12 weeks or longer at week 68 [19]. Faricimab received regulatory approval for retinal vein occlusion in the United States in 2023 and in Japan in March 2024, with subsequent international approvals expanding global availability [20].

Despite the encouraging results of BALATON and COMINO, the generalisability of pivotal trial findings to routine clinical practice remains uncertain, particularly in patients excluded from strict trial inclusion criteria such as those previously treated with other anti-VEGF agents who may benefit from switching therapy. A growing body of real-world evidence has emerged, including the multicentre FARI-LATAM case series [21] and a number of single-centre cohorts from Europe and East Asia, but these reports are heterogeneous in design, follow-up duration, loading regimen, and patient selection, and have not previously been synthesised. To date, no systematic review or meta-analysis has integrated the pivotal randomised evidence with the emerging real-world data to provide pooled estimates of effectiveness, treatment durability, and safety of intravitreal faricimab in macular oedema secondary to RVO. The aims of this systematic review and meta-analysis were therefore: (1) to estimate the pooled mean change in best-corrected visual acuity at 6 months following initiation of intravitreal faricimab, separately for treatment-naïve eyes and for eyes switched from prior anti-VEGF therapy; (2) to characterise treatment burden and durability by synthesising injection counts, treatment intervals, and proportions of eyes achieving extended dosing intervals; (3) to evaluate anatomical outcomes including changes in central retinal thickness and proportions of eyes achieving anatomical resolution; and (4) to summarise the safety profile of faricimab in this indication across pivotal trial and real-world settings.

## Methods

### Protocol and registration

This systematic review and meta-analysis was conducted in accordance with the Preferred Reporting Items for Systematic Reviews and Meta-Analyses (PRISMA) 2020 statement [22]. The protocol was initially developed and finalised on 18th April 2026 and was formally prospectively registered with the International Prospective Register of Systematic Reviews (PROSPERO; registration number CRD420261384239) on the 1st May 2026 [23].

### Eligibility criteria

Studies were included if they reported outcomes following intravitreal faricimab (6.0 mg) for macular oedema secondary to retinal vein occlusion. Eligible designs were randomised controlled trials, prospective and retrospective comparative cohorts, and single-arm cohort studies. Both treatment-naïve eyes and eyes switched to faricimab from prior anti-vascular endothelial growth factor (anti-VEGF) therapy were eligible. Switch cohorts were conceptualised within two clinical phenotypes: refractory switches (eyes switched from prior anti-VEGF therapy because of inadequate anatomical or functional response, including persistent or recurrent macular oedema and inability to extend treatment intervals) and durability switches (eyes switched from prior anti-VEGF therapy with stable disease control but with the aim of reducing treatment burden). Both phenotypes were eligible for inclusion. Studies enrolling fewer than 10 eyes, conference abstracts without full data, and studies of macular oedema secondary to causes other than retinal vein occlusion were excluded. No data or language restrictions were placed.

### Search strategy

PubMed, EMBASE, and the Cochrane Central Register of Controlled Trials were systematically searched from inception to 1st May 2026. Search terms comprised free-text terms for faricimab (including the brand name Vabysmo and the development codes RG-7716 and RO-6867461), retinal vein occlusion, macular oedema, branch retinal vein occlusion, central retinal vein occlusion, and hemiretinal vein occlusion. Reference lists of included studies and relevant systematic reviews were hand-searched. The search strategy was developed by the review team in consultation with the prespecified eligibility criteria documented in the registered protocol. The full search strategy is seen in supplementary file 3.

### Study selection and data extraction

Two reviewers (AA, SB) independently screened titles and abstracts, followed by full-text review of potentially eligible studies, with disagreements resolved by a third or fourth reviewer. Data extraction was performed in duplicate using a piloted standardised extraction form, with adjudication by a third reviewer where required. Extracted variables included study design, country, follow-up duration, baseline patient characteristics, prior treatment history, loading regimen, maintenance regimen, best-corrected visual acuity (BCVA), central subfield thickness (CST), injection counts, treatment intervals, and adverse events.

### Outcomes

The two co-primary outcomes were mean change in BCVA at 6 months (in Early Treatment Diabetic Retinopathy Study (ETDRS) letters) and mean number of intravitreal injections per month. Secondary outcomes were change in CST or central macular thickness, treatment interval at last follow-up, proportion of eyes achieving anatomical resolution (defined as absence of intraretinal or subretinal fluid on optical coherence tomography), proportion gaining 15 or more ETDRS letters, proportion achieving treatment intervals of 12 or more weeks and 16 or more weeks, and incidence of ocular and systemic adverse events.

### Risk of bias assessment

The Cochrane Risk of Bias 2 (RoB 2) tool [24] was applied to the BALATON and COMINO randomised comparative phase data. The Risk Of Bias In Non-randomised Studies of Interventions (ROBINS-I) tool [25] was applied to the comparative observational study and to all single-arm cohort studies treated as pre-post within-subject comparisons in line with Cochrane Handbook guidance. Two reviewers (AA, SB) independently assessed each study, with disagreements resolved by discussion or third/fourth reviewer adjudication. Risk-of-bias judgements were visualised using the robvis tool [26].

### Data harmonisation

Visual acuity values reported in logMAR were converted to ETDRS letters using the formula change in ETDRS = -50 × change in logMAR [27]. Where studies reported median and interquartile range, mean and standard deviation were estimated using the method described by Wan et al. [28]. Standard errors were converted to standard deviations using the relationship SD = SE × square root of n. Where 95% confidence intervals were reported, standard deviations were derived as (upper - lower) × square root of n / 3.92. For pre-post within-subject comparisons, the standard deviation of the change was derived assuming a baseline-final correlation of 0.5 in line with Cochrane Handbook recommendations (Section 6.5.2.8) [29].

### Statistical analysis

Random-effects meta-analysis was prespecified for the two primary BCVA outcomes using the inverse variance method with the DerSimonian-Laird estimator for between-study variance [30], with Hartung-Knapp-Sidik-Jonkman adjustment applied to confidence intervals [31] to account for the small number of pooled studies. The summary measure was the pooled mean change (MRAW) in ETDRS letters. Heterogeneity was assessed using the I-squared statistic, tau-squared, and the Cochran Q statistic. The 95% prediction interval was calculated to represent the expected range of true effects in a comparable future study. Analyses were performed using metanalysisonline.com [32]. Outcomes for which fewer than three studies provided sufficient data, or where definitional or methodological heterogeneity precluded pooling, were synthesised narratively. Certainty of evidence was assessed per outcome using the Grading of Recommendations, Assessment, Development and Evaluation (GRADE) framework adapted for systematic reviews of mixed evidence streams [33].

## Results

### Study selection

**Fig. 1:**
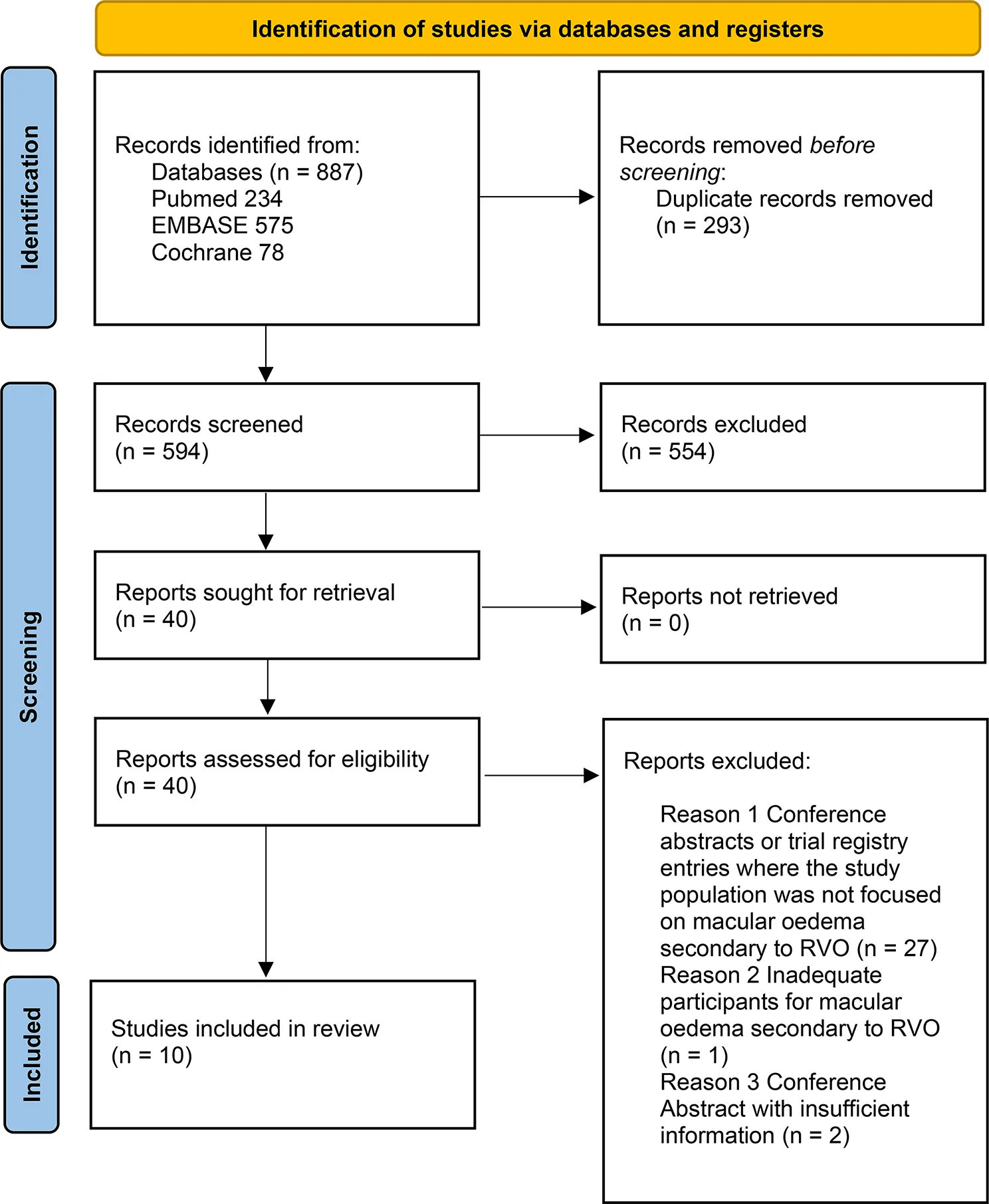
PRISMA 2020 flow diagram showing the identification, screening, eligibility, and inclusion of studies in this systematic review and meta-analysis

A total of 887 records were identified through systematic searches of PubMed (*n* = 234), EMBASE (*n* = 575), and the Cochrane Library (*n* = 78). Following removal of 293 duplicate records, 594 records were screened by title and abstract, of which 554 were excluded. Forty reports were sought for retrieval and assessed for eligibility against the prespecified inclusion criteria. Thirty reports were excluded with reasons: 27 records identified through EMBASE and Cochrane CENTRAL searches were conference abstracts or trial registry entries describing retinal disease populations not specific to macular oedema secondary to retinal vein occlusion, one record described a population with inadequate proportion of participants with macular oedema secondary to retinal vein occlusion; and two records were conference abstracts with insufficient information for data extraction. Ten studies met all eligibility criteria and were included in the final synthesis (Fig. 1).

### Study characteristics

The 10 included studies, summarised in Table 1, comprised one study containing the two randomised controlled trials (BALATON and COMINO; Danzig et al. [19] with linked week-24 comparative data from Tadayoni et al. [18]) and nine non-randomised studies. For the purposes of this synthesis, the BALATON and COMINO trials (Danzig et al. [19] with linked week-24 data from Tadayoni et al. [18]) are designated as the randomised trial evidence, while the nine non-randomised studies (Hafner et al. [34], Hikichi et al. 2025 [35], Hikichi et al. 2026 [36], Hirakata et al. [37], Inokuchi et al. [38], Kilani et al. [39], Sun et al. [40], Wolfrum et al. [41], and Wu et al. [21]) are designated as the real-world evidence stream. The non-randomised evidence comprised one prospective cohort [39], one retrospective comparative observational study [36], one retrospective multicentre case series (FARI-LATAM [21]), and five retrospective single-centre cohorts [34, 35, 37, 40, 41]. Studies were conducted across multiple countries including Germany, Japan, China, and Latin American sites, with the BALATON and COMINO trials enrolling patients from 22 countries across North America, Europe, Asia, and South America. A total of 1,620 eyes from approximately 1,500 patients were included across the 10 studies. Treatment-naïve cohorts were reported by four studies (Danzig et al. BALATON and COMINO [19], Hikichi et al. [36], and the naïve subgroups of Wu et al., Hirakata et al., and Inokuchi et al. [21, 37, 38]), while previously-treated switch cohorts were reported by eight studies. Follow-up duration ranged from 11.7 weeks [39] to 72 weeks [19], with the majority of real-world cohorts reporting outcomes between three and nine months following initiation of intravitreal faricimab. Loading regimens varied substantially across studies, ranging from six monthly fixed dosing followed by modified treat-and-extend (BALATON and COMINO) to single loading dose plus pro re nata [36, 37] to physician-discretion variable approaches [21].

**Table 1:** Study characteristics

Field	Danzig et al. 2025 (BALATON)	Danzig et al. 2025 (COMINO)	Hafneret al. 2025	Wu et al. 2026 (FARI-LATAM)	Inokuchi et al. 2025	Kilani et al. 2026	Hirakata et al. 2025	Hikichi et al. 2026	Hikichi et al. 2025	Sun et al. 2025	Wolfrum et al. 2026
Country	Multinational	Multinational	Germany (Munich)	Latin America (multi)	Japan (Yokohama)	Germany (Ulm)	Japan (Tokyo)	Japan (Sapporo)	Japan (Sapporo)	China (Beijing)	Germany (Mainz)
Centres	Multicentre	Multicentre	Single	Multicentre	Single	Single	Single	Single	Single	Single	Single
Design	Phase III RCT (Pt 1) + single-arm extension (Pt 2)	Phase III RCT (Pt 1) + single-arm extension (Pt 2)	Retrospective cohort	Retrospective multicentre case series	Retrospective cohort	**Prospective** cohort	Retrospective cohort	Retrospective cohort	Retrospective cohort	Retrospective cohort	Retrospective cohort
Stream	RCT comparative (wk 0–24) + single-arm (wk 24–72)	RCT comparative (wk 0–24) + single-arm (wk 24–72)	Single-arm switch	Single-arm × 3 RVO subgroups	Single-arm mixed	Single-arm switch	Single-arm naïve + switch	Comparative observational + single-arm	Single-arm switch	Single-arm switch	Single-arm switch
N eyes	553 (276 fari / 277 afli)	729 (366 fari / 363 afli)	19	70 RVO (29 naïve CRVO + 21 prior-tx CRVO + 20 prior-tx BRVO)	23	27	30 (17 naïve / 13 switch)	54 (25 fari / 29 afli)	42	52	21
N patients	553 (1 eye/pt)	729 (1 eye/pt)	19	~ 70	23	27 (1 eye/pt explicit)	30	54 (first eye only)	42 (first eye only)	52	20 (1 patient bilateral)
Mean age (yrs)	Reported in Tadayoni 2024	Reported in Tadayoni 2024	64.8 ± 12.7	Naïve CRVO 71.8; PT CRVO 71.6; PT BRVO 68.2	Median 81 (IQR 75.5–86); mean 79.6	70.5 ± 11.4	Naïve 69.0 ± 9.9; Switch 64.0 ± 6.7 (*p* = 0.001)	68.3 ± 11.4	74.1 ± 1.4 (SE)	63.05 ± 12.41	68.3 ± 11.7
Sex (M/F)	Reported in Tadayoni 2024	Reported in Tadayoni 2024	11/8	Not reported per RVO subgroup	10/13	17/10	16/14	30/24	19/23	19/33	10/11
RVO subtype	BRVO 100%	CRVO + HRVO	BRVO/CRVO mix - proportions NOT reported (gap)	Naïve CRVO subgroup; PT CRVO subgroup; PT BRVO subgroup	BRVO 17 (73.9%), CRVO 6 (26.1%)	CRVO 12 (44%), BRVO 15 (56%)	Naïve: BRVO 13, CRVO 4; Switch: BRVO 7, hemi-CRVO 2, CRVO 4	BRVO 100% (33 major / 21 macular)	BRVO 23 (54.8%), CRVO 17 (40.5%), hemi-CRVO 2 (4.8%)	BRVO 18 (34.6%), CRVO 34 (65.4%)	BRVO 17 (81%), CRVO 4 (19%)
Treatment status	100% naïve	100% naïve	100% pretreated; 14/19 anti-VEGF, 5/19 steroid	Mixed (3 separate subgroups)	Naïve 5 (21.7%), pretreated 18 (78.3%)	100% pretreated	Naïve 17 + Switch 13	100% naïve	100% pretreated	100% pretreated	100% pretreated
Mean prior anti-VEGF (switch/PT eyes)	N/A	N/A	33.2 ± 26.1	Naïve CRVO 0; PT CRVO 8.8; PT BRVO 10.7	Median 10.5 (IQR 9–17) non-naïve	Median 23 (IQR 15–56)	Switch: 16.2 ± 12.3	N/A	9.8 ± 1.4 (SE)	9.16 ± 4.26	36.2 ± 25.0
Prior dexamethasone implant	Excluded	Excluded	1.4 ± 3.4 (5 eyes most recent)	Some included	Not reported	Excluded	Not reported	Excluded	Excluded	18/52 (34.6%) had ≥ 1 implant	4/21 (19%) most recent
Refractoriness criteria (switch)	N/A	N/A	Persistent IRF after ≥ 3 monthly anti-VEGF, OR recurrence on extension >6wk; or steroid criteria	Physician judgement, “poor response”	Not formally defined	Persistent IRF + CST ≥ 270 μm despite ≥ 3 anti-VEGF at ≤6wk	≥ 3 prior anti-VEGF + persistent fluid OR cannot extend >8wk OR cannot stop after >2y	N/A	Cannot extend interval to ~3mo despite ≥ 3 anti-VEGF on PRN	≥ 5 anti-VEGF in 12mo OR ≥ 4 in 6mo, CMT ≥ 300 μm, residual SRF/IRF	Persistent SRF/IRF or < 5% CST reduction after monthly series, or 3mo after steroid
Loading regimen	6 monthly Q4W → modified T&E	6 monthly Q4W → modified T&E	4 monthly → T&E	Variable (physician discretion)	Effectively 1 (78% had only 1 injection)	1 + modified T&E (single loading dose only)	1 + PRN	1 + PRN	No re-loading (continued T&E)	PRN	3 monthly → PRN
Maintenance regimen	Modified T&E Q4W↔Q16W; stricter than YOSEMITE/RHINE	Modified T&E Q4W↔Q16W	T&E individualised	Variable	PRN	T&E ±2wk increments, min Q4W	PRN (re-inject if fluid on OCT)	PRN (re-inject if any IRF)	T&E ±2wk increments	PRN	PRN
Follow-up	72 weeks	72 weeks	8.9 ± 1.3 months (≥ 9mo required)	Naïve CRVO 26.2wk; PT CRVO 21.8wk; PT BRVO 28.1wk	3 months (≥ 3mo required)	11.7 ± 2.8 weeks (range 9–17)	Naïve 3.7 ± 2.7mo; Switch 4.9 ± 2.9mo	6 months	8.3 ± 0.3 months (range 6–12)	25.65 ± 11.87 weeks	~ 12 weeks (4wk after 3rd injection)
OCT device	SD or SS-OCT, central reading	SD or SS-OCT, central reading	Spectralis HRA + OCT	Spectralis / Cirrus / Optovue / Topcon	SD-OCT (device not specified)	Spectralis HRA2 + OCT or ZEISS CIRRUS 5000	Cirrus HD-OCT or Spectralis	Zeiss OCT 3000 / Canon Xephilio	Cirrus 5000 / Canon Xephilio	SS-OCT + UW-OCTA (Intalight)	Spectralis (manual segmentation verified)
BCVA method	ETDRS at 4 m	ETDRS at 4 m	Snellen 4 m → logMAR	Snellen → logMAR	logMAR (likely Landolt)	Snellen decimal → logMAR	logMAR (likely Landolt)	Landolt → logMAR	Landolt → logMAR	Snellen → ETDRS letters	Decimal VA → logMAR via Holladay 1997
Outcome format	Adjusted mean + 95.03% CI (MMRM)	Adjusted mean + 95.03% CI (MMRM)	Median + IQR	Mean ± 95% CI	Median + IQR	Median + IQR	Mean ± SD	Mean ± SD	Mean ± **SE** (conversion needed)	Mean ± SD	Mean ± SD
Bilateral patients	None	None	None	Some (757 eyes/732 pts overall)	None	None	None	None	None	None	1 patient

### Risk of bias assessment

**Fig. 2:**
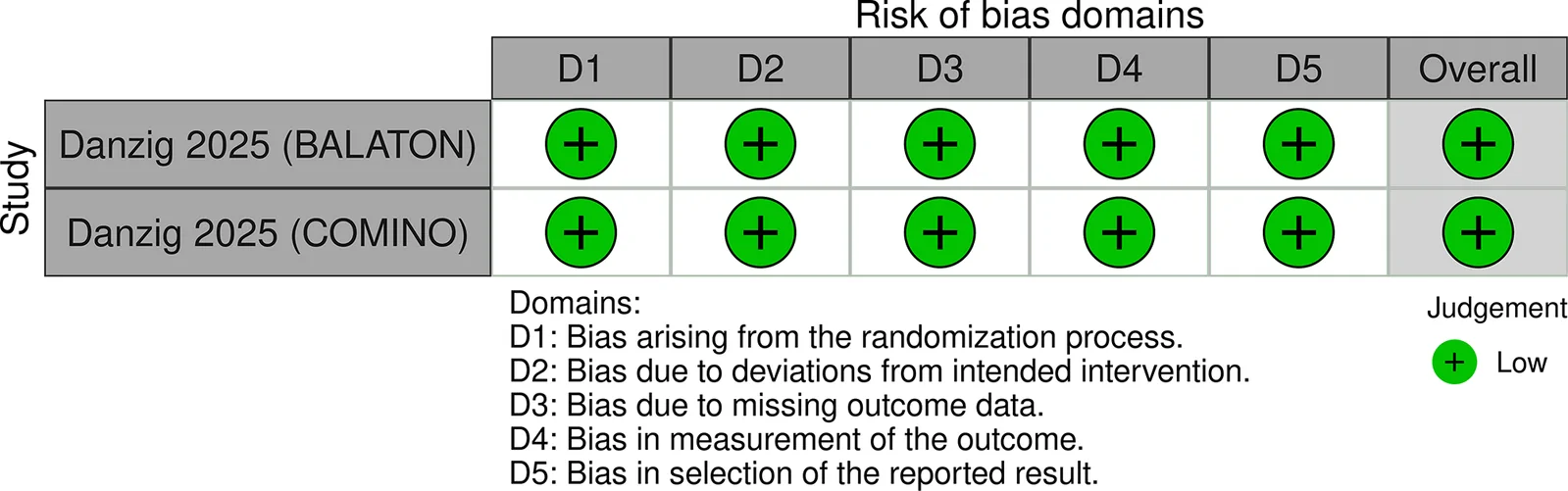
Cochrane risk of bias 2 (RoB 2) traffic light plot showing per-domain and overall risk-of-bias judgements for randomised trials (BALATON and COMINO)

**Fig. 3:**
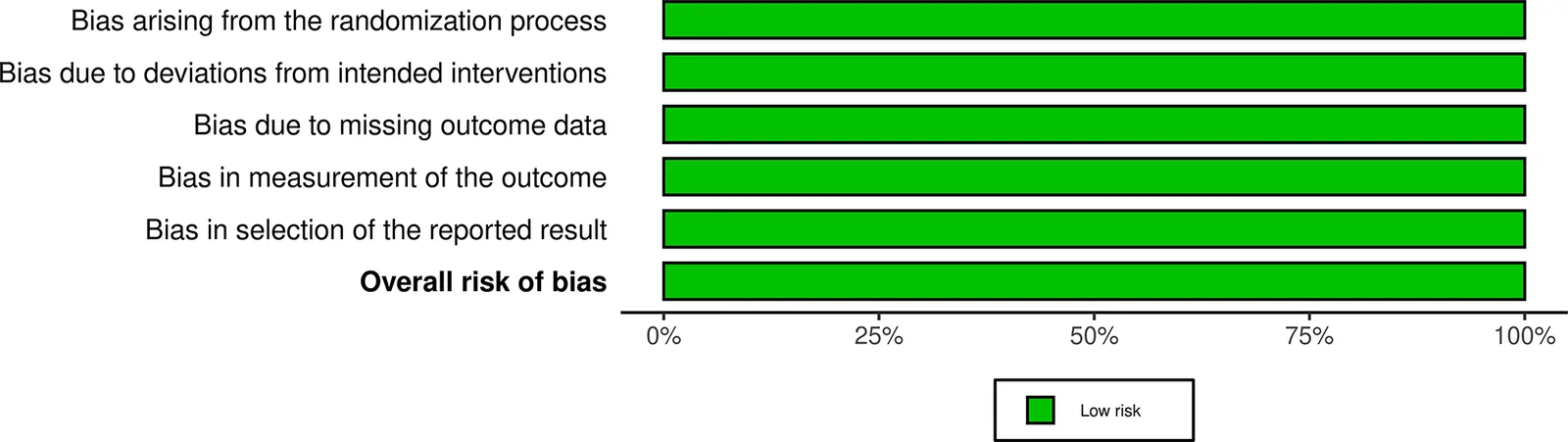
Cochrane risk of bias 2 (RoB 2) summary plot showing the proportion of randomised studies at each level of risk of bias across the five RoB 2 domains

**Fig. 4:**
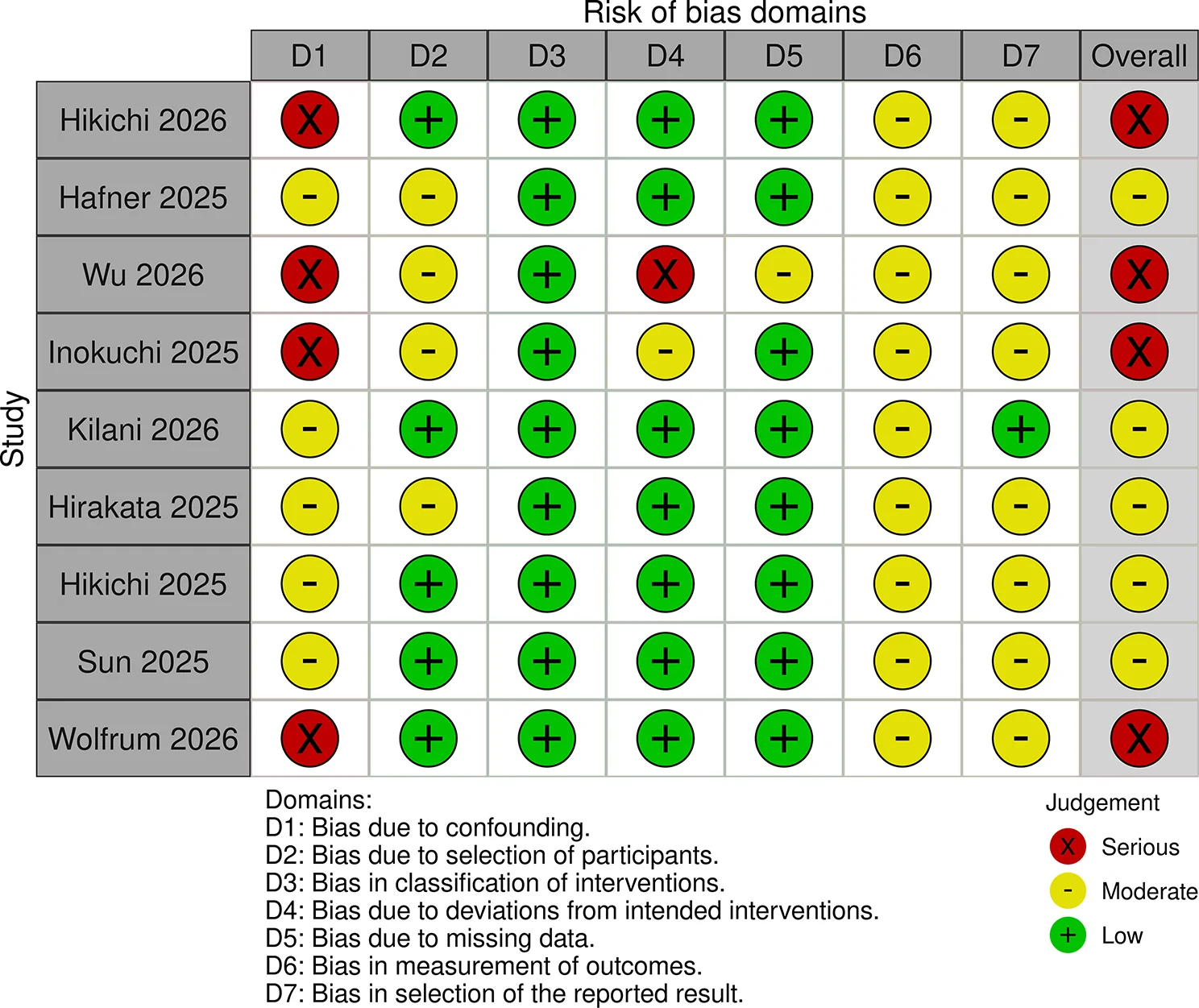
Risk of bias in non-randomised studies of interventions (ROBINS-I) traffic light plot showing per-domain and overall risk-of-bias judgements for the nine non-randomised studies included in this review

**Fig. 5:**
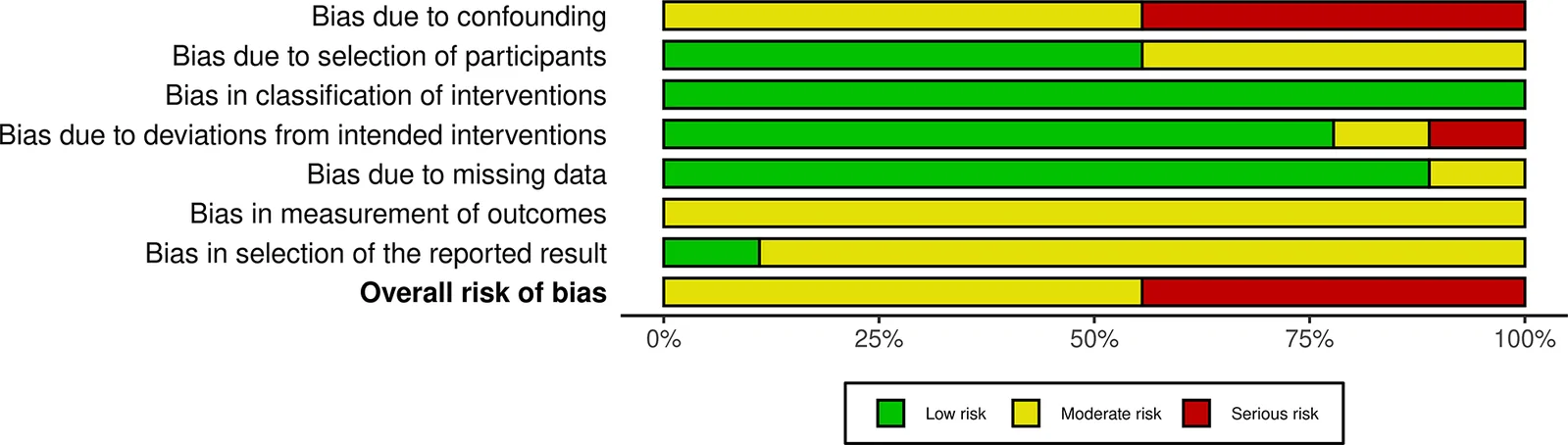
Risk of bias in non-randomised studies of interventions (ROBINS-I) summary plot showing the proportion of non-randomised studies at each level of risk of bias across the seven ROBINS-I domains

The two phase III randomised trials contributing comparative data (BALATON and COMINO) [19] were judged at low risk of bias across all five RoB 2 domains, supported by central masked outcome adjudication, intention-to-treat analysis with prespecified sensitivity analyses for missing data, and adherence to a registered protocol (Figs. 2 and 3). Among the nine non-randomised studies, four were judged at serious and five at moderate overall risk of bias. The dominant source of bias was confounding, particularly in single-arm switch cohorts where pre-post within-subject comparisons did not address regression to the mean or natural disease history, and where extreme prior treatment burden [41], heterogeneous physician-discretion loading regimens [21], or substantial undertreatment [38] amplified this concern. Measurement of outcomes was judged at moderate risk in all non-randomised studies because of unmasked retrospective chart review (Figs. 4 and 5).

### Best corrected visual acuity

#### Pooled effect of faricimab on best-corrected visual acuity at 6 months

##### Treatment-naïve eyes (Fig. 6 and Fig. 7)

Four contributions from three studies provided BCVA change data at approximately 6 months (Danzig et al. [19] BALATON faricimab arm, *n* = 276; Danzig et al. COMINO faricimab arm, *n* = 366; Hikichi et al. [36] faricimab arm, *n* = 25; Wu et al. [21] naïve CRVO subgroup, *n* = 29; total *N* = 696). The random-effects pooled mean BCVA change with Hartung-Knapp-Sidik-Jonkman adjustment was + 16.89 ETDRS letters (95% CI 16.05 to 17.72; I² = 0.0%; τ² = 0; Q = 0.96, df = 3, *p* = 0.81; 95% prediction interval 15.41 to 18.36).

A sensitivity analysis (Fig. 7) excluding the BALATON and COMINO randomised arms was conducted using the two real-world treatment-naïve contributions (Hikichi et al. [36] faricimab arm, *n* = 25; Wu et al. [21] naïve CRVO subgroup, *n* = 29; total *N* = 54), applying the same random-effects inverse-variance method with Hartung-Knapp-Sidik-Jonkman adjustment as the primary analysis. The pooled mean BCVA change was + 16.48 ETDRS letters (95% CI − 14.58 to + 47.53; I² = 0.0%; Cochran Q = 0.94, df = 1, *p* = 0.33; 95% prediction interval − 15.60 to + 48.55).

##### Switch cohorts (Fig. 8)

Four contributions from three studies provided BCVA change data at approximately 6 months (Wu et al. [21] pretreated CRVO subgroup, *n* = 21; Wu et al. pretreated BRVO subgroup, *n* = 20; Sun et al. [40], *n* = 52; Hirakata et al. [37] switch cohort, *n* = 13; total *N* = 106). The random-effects pooled mean BCVA change was + 8.73 ETDRS letters (95% CI 4.75 to 12.71; I² = 0.0%; τ² = 0; Q = 1.42, df = 3, *p* = 0.70; 95% prediction interval 2.94 to 14.51).

### Studies not included in primary pooled analyses

Four studies were excluded from the BCVA forest plots on prespecified timepoint grounds (follow-up outside the 4.5-to-7.5-month window) and reported narratively. Hafner et al. [34] (*n* = 19; switch cohort; FU 9 months) reported median logMAR BCVA improvement from 0.10 to 0.00 in the anti-VEGF pretreated subgroup, with median treatment interval increasing from 6.5 to 8.07 weeks (*p* = 0.01). Hikichi et al. [35] (*n* = 42; switch cohort; FU 8.3 months) reported significant logMAR improvement from 0.16 ± 0.03 (SE) to 0.02 ± 0.02 (*p* < 0.01) and treatment interval extension from 12.3 to 16.2 weeks (*p* < 0.01). Wolfrum et al. [41] (*n* = 21; heavily pretreated switch cohort; FU 3 months) reported a mean BCVA change of + 5 letters (*p* = 0.257) and central retinal volume reduction (median Δ − 14 mm³; *p* = 0.011), with 33.3% achieving complete retinal dryness. Kilani et al. [39] (*n* = 27; switch cohort; FU 11.7 weeks) reported median logMAR improvement from 0.2 to 0.1 (*p* = 0.022) and treatment interval extension from 4.6 to 7.3 weeks (*p* < 0.001). Inokuchi et al. [38] (*n* = 23; mixed cohort; FU 3 months) reported median logMAR change of 0.12 in the non-naïve subgroup (*p* = 0.014), with 52.2% achieving complete fluid resolution; 78.3% of patients received only a single faricimab injection during the follow-up period. The Hirakata et al. [37] treatment-naïve subgroup (*n* = 17; FU 3.7 months) reported logMAR improvement from 0.30 ± 0.37 to 0.11 ± 0.20 (*p* = 0.01).

All studies reported BCVA changes directionally consistent with the pooled estimates [19, 21, 34–41]. No included study reported a net loss of vision in any analytic subgroup [19, 21, 34–41].

### Treatment burden and durability

**Table 2:** Injection counts and rates across included studies

Study	Year	*n*	Population	Loading regimen	Total injections (mean +/- SD)	Follow-up (months)	Injection rate (per month)
Danzig et al. (BALATON wk 24–72)	2025	276	Naive BRVO, faricimab arm	6 monthly Q4W then modified T&E	4.8 +/- 2.6	11.1	0.43
Danzig et al. (BALATON wk 24–72)	2025	277	Naive BRVO, prior aflibercept	6 monthly Q4W then modified T&E	4.9 +/- 2.8	11.1	0.44
Danzig et al. (COMINO wk 24–72)	2025	366	Naive CRVO/HRVO, faricimab arm	6 monthly Q4W then modified T&E	5.6 +/- 3.0	11.1	0.50
Danzig et al. (COMINO wk 24–72)	2025	363	Naive CRVO/HRVO, prior aflibercept	6 monthly Q4W then modified T&E	5.5 +/- 2.9	11.1	0.50
Hikichi et al. (faricimab arm)	2026	25	Naive BRVO	1 + PRN	1.96 +/- 0.68	6.0	0.33
Hikichi et al. (aflibercept arm)	2026	29	Naive BRVO	1 + PRN	2.45 +/- 0.87	6.0	0.41
Hirakata et al. (naive)	2025	17	Naive mixed	1 + PRN	1.4 +/- 0.7	3.7	0.38
Wu et al. (naive CRVO)	2026	29	Naive CRVO	Variable physician discretion	3.7 (95% CI 2.6 to 4.8)	6.0	0.62
Wu et al. (pretreated CRVO)	2026	21	Switch CRVO	Variable physician discretion	3.4 (SD not reported)	5.0	0.68
Wu et al. (pretreated BRVO)	2026	20	Switch BRVO	Variable physician discretion	3.5 (SD not reported)	6.5	0.54
Hirakata et al. (switch)	2025	13	Switch mixed	1 + PRN	2.4 +/- 2.1	4.9	0.49
Sun et al.	2025	52	Switch mixed	PRN throughout	2.97 +/- 0.74	5.9	0.50
Inokuchi et al.	2025	23	Mixed	Effectively 1 (78% had only 1 injection)	1.4 (range 1 to 3)	3.0	0.47
Wolfrum et al.	2026	21	Heavily pretreated switch	3 monthly (fixed protocol)	3 (protocol-fixed)	3.0	1.00
Kilani et al.	2026	27	Switch	1 + protocol extensions	3 (protocol-fixed)	2.7	1.11
Hafner et al.	2025	19	Switch (anti-VEGF or steroid pretreated)	4 monthly then T&E	Not directly tabulated	9.0	Not calculable
Hikichi (switch) et al.	2025	42	Switch mixed	No re-loading (continued T&E)	Not directly tabulated	8.3	Not calculable

**Table 3:** Treatment interval data across included studies

Study	Year	*n*	Population	Pre-faricimab interval (weeks)	Post-faricimab interval (weeks)	Mean change (weeks)	*p*-value	Q12W or longer at final visit	Q16W or longer at final visit
Hikichi et al. (switch)	2025	42	Switch mixed	12.3 +/- 2.59	16.2 +/- 3.24	+ 3.9	< 0.01	Not separately reported	Not separately reported
Hafner et al. (anti-VEGF pretreated)	2025	14	Switch	6.5 (median)	8.07 (median, month 9)	+ 1.57 (median)	0.01	21.4%	0%
Sun et al.	2025	52	Switch mixed	Not reported	14.67 +/- 6.42	Not calculable	Not applicable	42.31%	30.77%
Kilani et al.	2026	27	Switch	4.6 +/- 0.8	7.3 +/- 1.6	+ 2.7	< 0.001	Not reported	Not reported
Wu et al. (naive CRVO)	2026	29	Naive CRVO	Not applicable (naive)	8.8 (95% CI 5.6 to 11.9)	Not applicable	Not applicable	Not separately reported	Not reported
Wu et al. (pretreated CRVO)	2026	21	Switch CRVO	Not reported	11.0 (95% CI 7.5 to 14.5)	Not calculable	Not applicable	Not separately reported	Not reported
Wu et al. (pretreated BRVO)	2026	20	Switch BRVO	Not reported	11.1 (95% CI 6.6 to 15.6)	Not calculable	Not applicable	Not separately reported	Not reported
Danzig et al. (BALATON, week 68)	2025	248	Naive, prior faricimab	Not applicable	Distribution at week 68	Not applicable	Not applicable	64%	52%
Danzig et al. (BALATON, week 68)	2025	244	Naive, prior aflibercept	Not applicable	Distribution at week 68	Not applicable	Not applicable	57%	48%
Danzig et al. (COMINO, week 68)	2025	330	Naive, prior faricimab	Not applicable	Distribution at week 68	Not applicable	Not applicable	45%	37%
Danzig et al. (COMINO, week 68)	2025	315	Naive, prior aflibercept	Not applicable	Distribution at week 68	Not applicable	Not applicable	50%	39%

Mean injection counts and rates of intravitreal faricimab administration across the included studies are summarised in Table 2. Treatment interval data are summarised in Table 3. Pooled meta-analysis was not undertaken for either metric because of substantial heterogeneity in loading regimens (six distinct loading approaches across the included studies, ranging from six monthly fixed dosing to single loading dose to PRN throughout), variation in follow-up duration (2.7 to 17 months) [19, 21, 34–41], and incomplete reporting of measures of variance for injection rates in several studies.

In treatment-naïve eyes, the BALATON and COMINO trials reported a median of 4 to 5 faricimab injections during the week 24 to 72 modified treat-and-extend extension phase, with 64% of BALATON patients and 45% of COMINO patients on prior faricimab achieving Q12W or longer intervals at week 68. In the only direct comparison of faricimab and aflibercept in treatment-naïve eyes [36], faricimab was associated with significantly fewer injections than aflibercept over 6 months (1.96 ± 0.68 versus 2.45 ± 0.87; *p* = 0.024). In switch cohorts, all studies reporting both pre-switch and post-switch metrics showed numerically lower injection burden following the switch to faricimab. Hikichi et al. [35] reported a mean treatment interval increase of 3.9 weeks (12.3 to 16.2 weeks; *p* < 0.01) over 8.3 months, Sun et al. [40] reported a reduction in injection rate from approximately 1.02 to 0.50 injections per month, and Kilani et al. [39] reported a mean treatment interval increase of 2.7 weeks (4.6 to 7.3 weeks; *p* < 0.001). Hafner et al. [34] reported median treatment interval extension from 6.5 to 8.07 weeks at month 9 (*p* = 0.01). Sun et al. reported that 42.31% of switch eyes achieved Q12W or longer intervals and 30.77% achieved Q16W or longer at the visit immediately preceding the final injection.

### Anatomical outcomes

Central subfield thickness (CST), central macular thickness (CMT), and central retinal thickness (CRT) changes from baseline, together with proportions of eyes achieving anatomical resolution (defined variably across studies as absence of intraretinal fluid, dry macula, or complete retinal dryness on optical coherence tomography), are summarised in Table 4. Pooled meta-analysis was not undertaken because of substantial heterogeneity in baseline severity (mean baseline CST/CMT ranged from 291 to 702 microns), variation in optical coherence tomography (OCT) device and segmentation method, and definitional differences in anatomical resolution endpoints [19, 21, 34–41].

**Table 4:** Anatomical outcomes across included studies

Study	Year	*n*	Population	Follow-up	Baseline CST/CMT/CRT (mean +/- SD, microns)	Final CST/CMT/CRT (microns)	Mean change (microns)	*p*-value	Anatomical resolution proportion	% change in CST/CMT/CRT from baseline
Danzig et al. (BALATON faricimab arm)	2025	276	Naive BRVO	Week 24	558.3 +/- 177.0	246.9 (adjusted)	-311.4 (95.03% CI -316.4 to -306.4)	Not directly tested	Not separately reported	-55.8
Danzig et al. (COMINO faricimab arm)	2025	366	Naive CRVO/HRVO	Week 24	702.2 +/- 244.0	240.6 (adjusted)	-461.6 (95.03% CI -471.4 to -451.9)	Not directly tested	Not separately reported	-65.7
Hikichi et al. (faricimab arm)	2026	25	Naive BRVO	6 months	598 +/- 106	192 +/- 57	-406	< 0.001	Not separately reported	-67.9
Hikichi et al. (aflibercept arm)	2026	29	Naive BRVO	6 months	635 +/- 161	223 +/- 80	-412	< 0.001	Not separately reported	-64.9
Wu et al. (naive CRVO)	2026	29	Naive CRVO	26.2 weeks	491 (95% CI 410 to 572)	287 (95% CI 229 to 344)	-204	0.0001	Not separately reported	--41.5
Hirakata et al. (naive)	2025	17	Naive mixed	3.7 months	442 +/- 117	313 +/- 78	-129	< 0.0001	70.6% (CMT < 300 microns 1 month after final injection)	-29.2
Wu et al. (pretreated CRVO)	2026	21	Switch CRVO	21.8 weeks	498 (95% CI 401 to 595)	347 (95% CI 266 to 428)	-151	Not separately tested	Not separately reported	-30.3
Wu et al. (pretreated BRVO)	2026	20	Switch BRVO	28.1 weeks	351 (95% CI 281 to 421)	274 (95% CI 236 to 312)	-77	0.018	Not separately reported	-21.9
Sun et al.	2025	52	Switch mixed	25.65 weeks	547.4 +/- 160.9	216.4 +/- 105.3	-331 (post-3rd injection)	< 0.001	78.85% (complete fluid resolution)	-60.5
Hirakata et al. (switch)	2025	13	Switch mixed	4.9 months	436 +/- 170	271 +/- 71	-165	0.0002	46.2% (CMT < 250 microns 1 month after final injection)	-37.8
Hikichi et al. (switch)	2025	42	Switch mixed	8.3 months	356 +/- 23 (SE)	205 +/- 4 (SE)	-151	< 0.01	64.3% (absence of intraretinal fluid)	-42.4
Hafner et al. (overall)	2025	19	Switch	9 months	325 (IQR 160)	285 (IQR 48, month 9)	-40 (median)	< 0.01	73.7% (absence of intraretinal fluid)	-12.3
Hafner et al. (anti-VEGF pretreated)	2025	14	Switch	9 months	310 (IQR 116)	282 (IQR 47, month 9)	-28 (median)	< 0.01	85.7% (absence of intraretinal fluid)	-9.0
Kilani et al.	2026	27	Switch	11.7 weeks	291 (IQR 281 to 325)	268 (IQR 253 to 289)	-23 (median)	< 0.001	48.1% (dry macula)	-7.9
Wolfrum et al.	2026	21	Heavily pretreated switch	~ 12 weeks	350.2 +/- 124.5	302.6 +/- 74.6	-47.6 (mean)	0.037	33.3% (complete retinal dryness)	-13.6
Inokuchi et al. (overall)	2025	23	Mixed	3 months	Not separately tabulated	Not separately tabulated	Not directly reported	Not applicable	52.2% (complete fluid resolution)	Not calculable
Inokuchi et al. (non-naive)	2025	18	Switch	3 months	343 (IQR 214.8 to 426.5)	210 (IQR 165.8 to 289.5)	Median trajectory − 133	0.0016	44.4% (complete fluid resolutio	-38.7 (median trajectory)

All studies reporting CST, CMT, or CRT outcomes documented statistically significant reductions following faricimab treatment, with no included study reporting net thickening at the final follow-up timepoint [19, 21, 34–41]. Treatment-naïve cohorts demonstrated larger absolute reductions, with mean changes ranging from − 129 microns (Hirakata et al. naïve subgroup) [37] to -461.6 microns (Danzig et al. [19] COMINO faricimab arm), reflecting both higher baseline severity and longer follow-up in the larger trial cohorts. Switch cohort reductions ranged from − 23 microns (11.7 weeks follow-up) [39] to -331 microns (25.65 weeks) [40]. Expressed as percentage change from baseline, central retinal thickness reductions ranged from approximately − 8% to − 68% across the included studies, with the largest relative reductions observed in treatment-naïve cohorts (range − 29% to − 68%) and more modest relative reductions in switch cohorts (range − 8% to − 60%) (Table 4). The Hikichi et al. [36] head-to-head comparison showed no significant difference between faricimab and aflibercept in CST reduction at 6 months in treatment-naïve BRVO eyes (-406 versus − 412 microns, both *p* < 0.001). Anatomical resolution proportions varied substantially across studies, ranging from 33.3% (complete retinal dryness at 12 weeks in heavily pretreated eyes) [41] to 85.7% (absence of intraretinal fluid in the anti-VEGF pretreated subgroup at month 9) [34], reflecting both follow-up duration and definitional differences. Among studies using the strictest definition of complete fluid resolution (no intraretinal or subretinal fluid), proportions ranged from 33.3% to 78.85%.

### Safety

Ocular and systemic adverse events reported across the included studies are summarised in Table 5. Pooled meta-analysis was not undertaken because of low overall event rates and substantial zero-event reporting across the real-world cohort studies, both of which limit the precision and interpretability of pooled proportions for rare events [19, 21, 34–41].

**Table 5:** Safety outcomes across included studies

Study	Year	*n* eyes	Follow-up	IOI events	Retinal vasculitis	RAO	Endophthalmitis	RVO worsening	APTC events
Danzig et al. (BALATON wk 0–24, faricimab arm)	2025	276	6 months	1 (0.4%)	0	0	0	1 (0.4%)	3 (1.1%)
Danzig et al. (BALATON wk 0–24, aflibercept arm)	2025	274	6 months	0	0	0	0	3 (1.1%)	4 (1.5%)
Danzig et al. (BALATON wk 24–72, faricimab maintenance)	2025	270	11 months	2 (0.7%)	0	0	0	9 (3.3%)	2 (0.7%)
Danzig et al. (BALATON wk 24–72, prior aflibercept switched to faricimab)	2025	267	11 months	3 (1.1%)	0	0	0	9 (3.4%)	8 (3.0%)
Danzig et al. (COMINO wk 0–24, faricimab arm)	2025	365	6 months	8 (2.2%)	0	3 (0.8%)	0	1 (0.3%)	4 (1.1%)
Danzig et al. (COMINO wk 0–24, aflibercept arm)	2025	361	6 months	4 (1.1%)	0	1 (0.3%)	1 (0.3%)	5 (1.4%)	5 (1.4%)
Danzig et al. (COMINO wk 24–72, faricimab maintenance)	2025	359	11 months	10 (2.8%)	0	2 (0.6%)	1 (0.3%)	11 (3.1%)	8 (2.2%)
Danzig et al. (COMINO wk 24–72, prior aflibercept switched to faricimab)	2025	342	11 months	5 (1.5%)	0	2 (0.6%)	0	8 (2.3%)	6 (1.8%)
Hafner et al.	2025	19	9 months	0	0	0	0	0	0
Wu et al. (RVO subset)	2026	70	5.0 to 6.5 months	1 (1.4%)	0	0	0	0	Not reported
Inokuchi et al.	2025	23	3 months	0	0	0	0	0	0
Kilani et al.	2026	27	11.7 weeks	0	0	0	0	0	0
Hirakata et al.	2025	30	3.7 to 4.9 months	0	0	0	0	0	0
Hikichi et al. (faricimab arm)	2026	25	6 months	0	0	0	0	0	0
Hikichi et al. (switch)	2025	42	8.3 months	0	0	0	0	0	0
Sun et al.	2025	52	25.65 weeks	0	0	0	0	0	0
Wolfrum et al.	2026	21	12 weeks	Not structured as outcome	Not structured as outcome	Not structured as outcome	Not structured as outcome	Not structured as outcome	Not structured as outcome

No retinal vasculitis was reported in any included study at any timepoint, across a combined denominator of 1,620 eye-treatments [19, 21, 34–41]. Intraocular inflammation events were concentrated in the BALATON and COMINO randomised trials, with the highest incidence observed in the COMINO faricimab arm (8 events; 2.2% during the week 0 to 24 comparative phase, increasing to a cumulative 18 events including the week 24 to 72 extension). Two of these events were serious uveitis cases considered drug-related by investigators, one of and were absent in all real-world which was associated with a sustained 24-letter loss of best-corrected visual acuity following 3 injections, accompanied by worsening cystoid macular oedema. The remaining IOI events were non-serious and resolved without permanent visual loss. Among the nine real-world cohort studies, only one IOI event was reported (an anterior uveitis in a pretreated CRVO eye, treated with topical steroids and resolved with discontinuation of faricimab) [21]. Retinal artery occlusion events occurred exclusively in the COMINO trial, with three events in the week 0 to 24 faricimab arm, two events in the week 24 to 72 faricimab maintenance group, two events in the prior-aflibercept-switched-to-faricimab group, and one event in the week 0 to 24 aflibercept arm [19]. One infectious endophthalmitis was reported in the COMINO aflibercept arm at week 0 to 24, associated with a 45-letter loss of vision that had not recovered at the time of primary analysis; one non-infectious endophthalmitis was reported in the COMINO faricimab maintenance arm [19]. RVO worsening events were reported in 0.3% to 3.4% of eyes per cohort across the BALATON and COMINO arms and were absent in all real-world cohort studies [19, 21, 34–40]. Anti-Platelet Trialists’ Collaboration (APTC) events, comprising non-fatal myocardial infarction, non-fatal stroke, and vascular death, ranged from 0.7% to 3.0% across the BALATON and COMINO sub-cohorts and were absent in all real-world cohort studies that reported this outcome [19, 34–40]. No included study reported any safety event leading to permanent vision loss in association with faricimab in a real-world setting [19, 21, 34–41].

### Certainty of evidence

The certainty of evidence for each primary and secondary outcome was assessed using the Grading of Recommendations, Assessment, Development and Evaluation (GRADE) framework adapted for systematic reviews of mixed evidence streams, with separate ratings for the comparative randomised evidence (Danzig et al. [19] BALATON and COMINO weeks 0 to 24) and the pooled real-world evidence (Table 6).

**Table 6:** GRADE summary of findings

Outcome	Studies (*n*)	Participants	Pooled estimate	Risk of bias	Inconsistency	Indirectness	Imprecision	Publication bias	Certainty
BCVA change at 6 months, treatment-naive eyes (single-arm pooled)	4 contributions, 3 studies	696	+ 16.89 ETDRS letters (95% CI 16.05 to 17.72)	Not serious (2 RCT arms low risk; 1 observational comparative serious; 1 single-arm observational moderate)	Not serious (I-squared = 0%)	Serious - Pooled estimate dominated by BALATON and COMINO randomised arms (96.6% of weight); limited real-world generalisability beyond the registration trial populations	Not serious (narrow CI)	Undetected	Moderate
BCVA change at 6 months, switch cohorts (single-arm pooled)	4 contributions, 3 studies	106	+ 8.73 ETDRS letters (95% CI 4.75 to 12.71)	Serious (all contributions from non-randomised cohorts; ROBINS-I serious or moderate)	Not serious (I-squared = 0%)	Serious - substantial heterogeneity in prior treatment intensity across contributing studies (mean prior anti-VEGF injection counts ranging from approximately 8.8 to 36.2), limiting transportability of the pooled estimate to any specific prior-treatment intensity	Serious (small N; wide prediction interval 2.94 to 14.51)	Undetected	Very low
Mean injections per month, treatment-naive eyes (faricimab vs. aflibercept)	1 study	54	0.327 vs. 0.408 inj/mo (*p* = 0.024)	Serious (single non-randomised observational study)	Not applicable (single study)	Not serious	Serious (single study, small N)	Undetected	Very low
Treatment interval extension, switch cohorts (narrative)	4 studies	134	Mean increase 1.6 to 3.9 weeks across studies	Moderate (ROBINS-I moderate to serious)	Not assessable (narrative)	Not serious	Serious (small individual study sizes)	Undetected	Low
Anatomical resolution proportion (narrative)	7 studies	222	33.3% to 85.7% (definitional heterogeneity)	Moderate (ROBINS-I moderate to serious)	Serious (definitional heterogeneity across studies)	Not serious	Serious (small individual study sizes)	Undetected	Very low
Intraocular inflammation, faricimab in real-world cohorts	8 studies	286	1 event in 286 eye-treatments (0.3%)	Moderate (ROBINS-I moderate to serious)	Not serious	Not serious	Serious (rare event, small total N for real-world cohorts)	Undetected	Low
Retinal vasculitis, all studies	10 studies	Approximately 1,620 eyes	0 events	Not serious (consistent across stream and timepoints)	Not serious (uniform zero across studies)	Not serious	Not serious	Undetected	Moderate

The certainty of evidence ranged from moderate to very low. The moderate certainty rating for the treatment-naïve BCVA outcome reflects the methodological strengths of the BALATON and COMINO randomised arms (low risk of bias, narrow confidence interval, negligible heterogeneity with I² = 0%), tempered by indirectness arising from the dominance of these registration trial contributions (96.6% of pooled weight) and consequent limited real-world generalisability of the synthesis. The very low certainty rating for switch cohort BCVA reflects the absence of randomised evidence in this stream, the small total sample size, the wide 95% prediction interval, and substantial heterogeneity in prior treatment intensity across contributing studies (mean prior anti-VEGF injection counts ranging from approximately 8.8 to 36.2). Treatment burden and anatomical outcomes were rated low to very low because of inconsistent reporting conventions, definitional heterogeneity, and small individual study sizes. The absence of retinal vasculitis was rated moderate certainty, reflecting consistent zero-event reporting across all 10 studies and approximately 1,620 eyes, despite the rare-event imprecision inherent in any null safety finding.

## Discussion

This systematic review and meta-analysis identified ten studies reporting outcomes of intravitreal faricimab for macular oedema secondary to retinal vein occlusion, comprising the BALATON and COMINO RCTs and nine non-randomised real-world cohorts. Pooled analysis of best-corrected visual acuity at approximately 6 months suggested a mean gain of + 16.89 ETDRS letters (95% CI 16.05 to 17.72) in treatment-naïve eyes and a mean gain of + 8.73 ETDRS letters (95% CI 4.75 to 12.71) in eyes switched to faricimab from prior anti-VEGF therapy, with negligible between-study heterogeneity (I² = 0% in both analyses). Treatment burden, anatomical outcomes, and safety could not be meta-analytically pooled because of methodological and definitional heterogeneity, but were synthesised narratively across the included studies. The narrative synthesis was consistent with treatment interval extension following initiation of or switch to faricimab, statistically significant reductions in central retinal thickness across all studies reporting this outcome, and a favourable short-term safety profile, with no retinal vasculitis events identified across the combined evidence base.

The pooled BCVA gain in treatment-naïve eyes (+ 16.89 ETDRS letters at approximately 6 months) appeared consistent with the registration trial estimates from BALATON and COMINO, in which faricimab achieved adjusted mean gains of approximately 17 letters at week 24 alongside non-inferiority to aflibercept [18], though this consistency primarily reflects the dominant contribution of the registration trial data to the pooled estimate. The pooled estimate was also consistent with the visual acuity gains reported in the pivotal trials of established anti-VEGF agents in this indication, including the CRUISE [6], BRIGHTER [7], VIBRANT [8], and COPERNICUS [9] studies, which reported 6-month BCVA gains in the range of 14 to 18 ETDRS letters in treatment-naïve cohorts. The pooled BCVA gain in switch cohorts (+ 8.73 ETDRS letters) was numerically smaller than the gain observed in treatment-naïve eyes, but appeared consistent with the more limited prior literature on switching between anti-VEGF agents in retinal vein occlusion, in which post-switch gains have generally ranged from approximately 5 to 10 letters depending on the prior treatment burden and refractoriness profile of the switching cohort [10, 14]. The narrative observation of treatment interval extension following switch to faricimab, with mean increases of approximately 2 to 4 weeks across the studies reporting this metric, may align with similar interval-extension findings reported for faricimab in neovascular age-related macular degeneration [15] and in real-world diabetic macular oedema cohorts, although direct comparison across indications should be made cautiously given differences in disease pathophysiology and treatment paradigms.

An important consideration when interpreting the switch cohort findings is the clinical phenotype of the eyes contributing to this evidence stream. Anti-VEGF switches in clinical practice typically reflect one of two rationales: inadequate response to prior therapy (refractory switches) or stable disease control with the goal of reducing treatment burden (durability switches). On review of the inclusion criteria and population characteristics of each included switch study, all nine switch contributions in this synthesis represent refractory phenotypes. The Wu et al. [21] pretreated CRVO and BRVO subgroups, Sun et al. [40] (“real-life. refractory macular edema secondary to retinal vein occlusion”), Hafner et al. [34] (“therapy-resistant macular edema”), Kilani et al. [39] (“anti-VEGF-refractory retinal vein occlusion”), and Wolfrum et al. [41] (heavily pretreated cohort with persistent disease) were explicitly framed as refractory in their respective publications. The Hikichi et al. [35] 2025 switch cohort enrolled eyes with relapsed macular oedema despite prior aflibercept treatment and an inability to extend treatment intervals beyond approximately three months. The Hirakata et al. [37] switch cohort documented inadequate response (*n* = 11) or ineffective prior therapy (*n* = 2) as the rationale for all 13 switches. The Inokuchi et al. [38] non-naïve subgroup also predominantly described inadequate prior response. The synthesis therefore characterises faricimab outcomes in refractory switch cohorts, with no durability-motivated switch cohort identified in the available literature. This phenotypic homogeneity supports the interpretability of the pooled switch estimate as reflecting a refractory clinical population, while acknowledging that the synthesis cannot speak to outcomes of faricimab in eyes switched purely for treatment burden reduction. The smaller magnitude of pooled BCVA gain in switch cohorts (+ 8.73 ETDRS letters) compared with treatment-naïve cohorts (+ 16.89 ETDRS letters) is therefore consistent with the established principle that prior anti-VEGF exposure with inadequate disease control may impose a functional ceiling on subsequent visual recovery, rather than reflecting a faricimab-specific limitation.

The pattern of approximately doubled visual gain in treatment-naïve compared with switch cohorts identified in this synthesis may reflect a functional ceiling effect arising from cumulative photoreceptor damage and chronic structural alteration in eyes previously exposed to prolonged anti-VEGF therapy [14]. Wolfrum et al. [41] explicitly framed their heavily pretreated cohort within this hypothesis, observing that only a minority of eyes (5 of 21) achieved meaningful functional improvement despite consistent anatomical response. A similar pattern of preserved anatomical response with attenuated functional gain has been reported in the TRUCKEE real-world cohort of treatment-experienced eyes with neovascular age-related macular degeneration [42], in which switching to faricimab yielded a modest mean BCVA improvement of 0.7 letters alongside a significant central subfield thickness reduction of 25 microns, suggesting that the structural-functional dissociation may be a class effect of switching late in the treatment course rather than a faricimab-specific limitation. The treatment durability findings of the present synthesis, particularly the Danzig et al. finding that approximately half of treatment-naïve eyes maintained 12-week or longer dosing intervals through week 68 [19], appear consistent with the durability profile observed in the pooled 2-year analysis of TENAYA and LUCERNE in nAMD, in which 78% of faricimab-treated eyes maintained Q12W or longer intervals at week 112 [43]. Real-world DME registries have similarly reported that approximately half of patients achieve extended dosing intervals on faricimab, with the recent FARWIDE-DMO study of 2,147 eyes from 35 UK National Health Service sites reporting mean injection numbers of 4.5 over 12 months in treatment-naïve eyes [44]. Taken together, these convergent findings across three indications may be consistent with the hypothesis that simultaneous Ang-2 and VEGF-A inhibition provides more durable vascular stabilisation than VEGF blockade alone [16, 17], although direct mechanistic confirmation across retinal vein occlusion specifically would require dedicated translational study.

There are several limitations of this review that should be acknowledged. The treatment-naïve forest plot was dominated by the BALATON and COMINO trials, which together contributed 96.6% of the pooled weight, and the pooled estimate therefore reflects the registration trial result with only minimal real-world contribution. The synthesis therefore provides robust confirmation of the registration trial findings but more limited independent confirmation of real-world effectiveness in treatment-naïve cohorts, and the GRADE certainty rating for this outcome has been set at Moderate rather than High to reflect this indirectness. The sensitivity analysis excluding the BALATON and COMINO contributions (Fig. 7) yielded a directionally consistent pooled point estimate (+ 16.48 ETDRS letters) but with a substantially wider confidence interval (− 14.58 to + 47.53) reflecting the small real-world sample size (*n* = 54), supporting the interpretation that the precision of the primary treatment-naïve estimate is driven by the registration trial data. The switch cohort meta-analysis included a modest total sample size (*n* = 106), with three of the four contributions using per-arm standard deviations derived from change-score confidence intervals and assumed within-arm variance distribution. The standard deviation of pre-post changes was derived assuming a baseline-final correlation of 0.5 in line with Cochrane Handbook guidance [29]. Three of the nine non-randomised studies originated from East Asian sites, which may limit generalisability across more diverse healthcare systems and ethnic populations. The majority of included real-world studies were retrospective chart reviews with relatively short follow-up durations (as short as 11.7 weeks in one cohort), and retrospective study designs are known to under-report rare adverse events; the absence of rare events such as retinal vasculitis in small, short-term retrospective cohorts should therefore not be interpreted as proof of safety, and ongoing pharmacovigilance through prospective registries and post-marketing surveillance is needed to detect rare adverse signals. Loading regimens, follow-up durations, and definitions of anatomical resolution varied substantially across the included studies, precluding meaningful pooled estimation of secondary outcomes. The synthesis characterises faricimab outcomes in refractory switch cohorts only, as no durability-motivated switch cohort was identified in the available literature; the effectiveness of faricimab in eyes switched from prior anti-VEGF therapy purely for treatment burden reduction therefore remains uncharacterised by this synthesis. A final methodological limitation was the search strategy which was developed by the review team but not peer-reviewed by an independent information specialist using the PRESS checklist; while the intervention terms used are highly specific (the proprietary drug name faricimab and its development codes), absence of independent search peer review is acknowledged as a methodological limitation that may have led to omission of relevant records.

The findings of this synthesis may have several implications for clinical practice and future research. The pooled BCVA estimates suggest that intravitreal faricimab is associated with substantial visual gains in treatment-naïve eyes and meaningful, though smaller, gains in switch cohorts, supporting its consideration as both a first-line and a switch option for macular oedema secondary to retinal vein occlusion. Several questions remain unanswered. The durability of faricimab beyond 12 months in real-world cohorts is not well characterised outside the BALATON and COMINO extension data, and longer-term standardised follow-up data would help to address this gap. The functional ceiling hypothesis raised by the differential gain magnitude between treatment-naïve and switch cohorts merits formal investigation, ideally through prospective protocolised switch studies capturing baseline structural biomarkers. The intraocular inflammation signal observed in the COMINO faricimab arm warrants ongoing safety surveillance, particularly in CRVO populations and heavily pretreated switch settings. Finally, head-to-head real-world comparative data between faricimab and aflibercept beyond the single Hikichi et al. cohort [36] would strengthen the evidence base for clinical decision-making, particularly where randomised comparative data are absent.

## Conclusion

This systematic review and meta-analysis synthesised evidence from ten studies evaluating intravitreal faricimab for macular oedema secondary to retinal vein occlusion. The synthesis supports faricimab as a therapeutic option in this indication, with the treatment-naïve pooled estimate primarily reflecting registration trial data and the switch cohort estimate characterising refractory phenotypes. Longer-term real-world cohorts, prospective protocolised studies of durability-motivated switches, and head-to-head comparative data with established anti-VEGF agents would strengthen the evidence base supporting clinical decision-making.

**Fig. 6:**
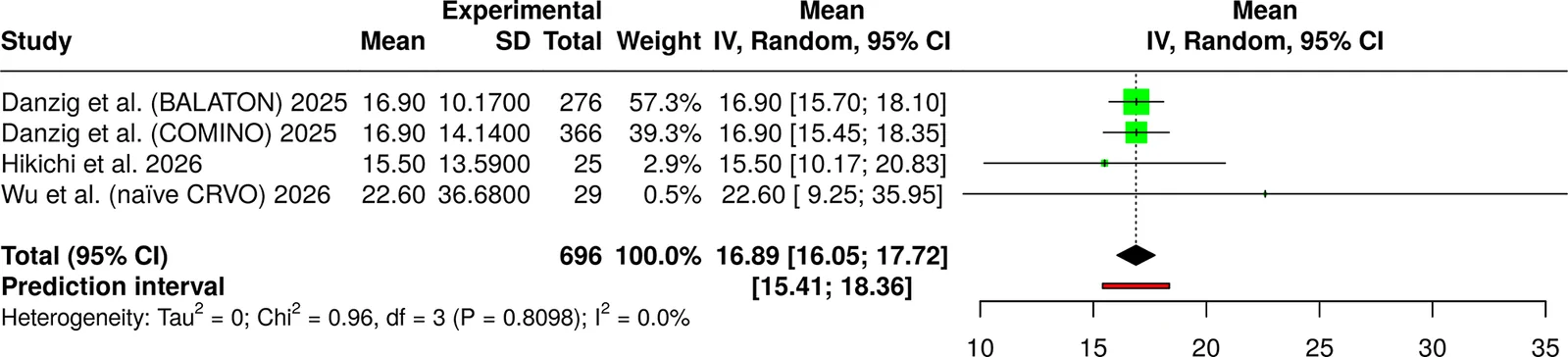
Random-effects meta-analysis forest plot of mean change in best-corrected visual acuity (Early Treatment Diabetic Retinopathy Study letters) at approximately 6 months following intravitreal faricimab in treatment-naïve cohorts

**Fig. 7:**
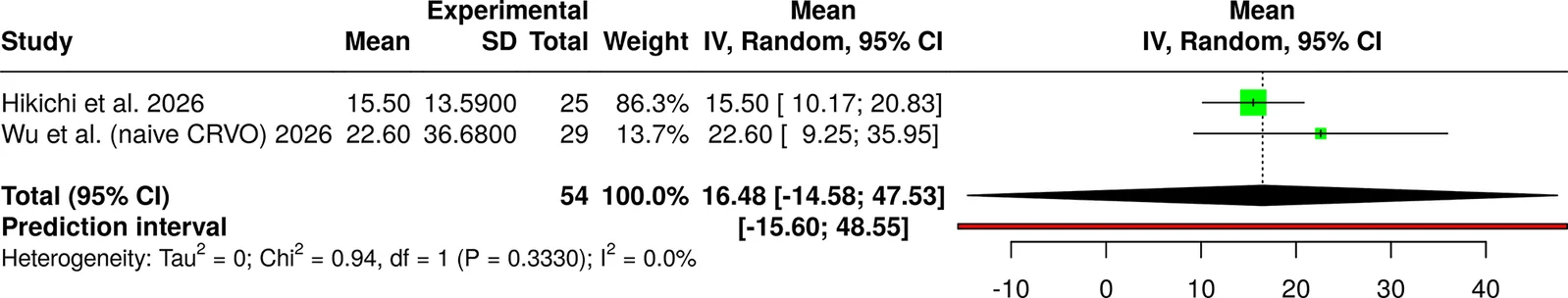
Sensitivity analysis: random-effects meta-analysis forest plot of mean change in best-corrected visual acuity (Early Treatment Diabetic Retinopathy Study letters) at approximately 6 months following intravitreal faricimab in treatment-naïve cohorts

**Fig. 8:**
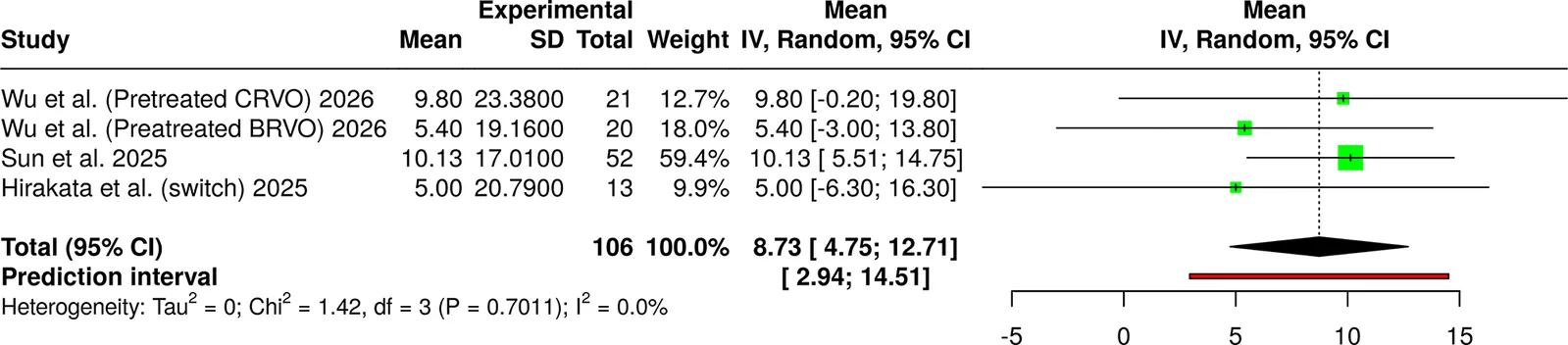
Random-effects meta-analysis forest plot of mean change in best-corrected visual acuity (Early Treatment Diabetic Retinopathy Study letters) at approximately 6 months following switch to intravitreal faricimab from prior anti-vascular endothelial growth factor therapy

## Supplementary Information

Below is the link to the electronic supplementary material.

Supplementary Material 1

Supplementary Material 2

Supplementary Material 3

## Data Availability

All data analysed during this systematic review are derived from the included studies, which are cited in the reference list and publicly available. The full data extraction table is provided as Supplementary File 2. The PRISMA 2020 checklist is provided as Supplementary File 1. Search strategy is provided in Supplementary file 3.

## Abbreviations

Ang-2: Angiopoietin-2
APTC: Anti-Platelet Trialists’ Collaboration (non-fatal myocardial infarction, non-fatal stroke, vascular death)
BCVA: Best-corrected visual acuity
BRVO: Branch retinal vein occlusion
CI: Confidence interval
CMT: Central macular thickness
CRT: Central retinal thickness
CRVO: Central retinal vein occlusion
CST: Central subfield thickness
ETDRS: Early Treatment Diabetic Retinopathy Study
GRADE: Grading of Recommendations, Assessment, Development and Evaluation
HKSJ: Hartung-Knapp-Sidik-Jonkman
HRVO: Hemiretinal vein occlusion
IOI: Intraocular inflammation
logMAR: Logarithm of the minimum angle of resolution
MD: Mean difference
MMRM: Mixed-model repeated measures
MRAW: Summarised raw means
OCT: Optical coherence tomography
PRISMA: Preferred Reporting Items for Systematic Reviews and Meta-Analyses
PROSPERO: International Prospective Register of Systematic Reviews
PRN: Pro re nata
RAO: Retinal artery occlusion
RoB 2: Cochrane Risk of Bias 2
ROBINS-I: Risk Of Bias In Non-randomised Studies of Interventions
RVO: Retinal vein occlusion
SD: Standard deviation
SE: Standard error
T&E: Treat-and-extend
VEGF: Vascular endothelial growth factor
