# Supplementary material for: Effectiveness and safety of intravitreal faricimab for macular oedema secondary to retinal vein occlusion: a systematic review and meta-analysis
Source: Int J Retina Vitreous. 2026 Jul 9;12:134. doi: 10.1186/s40942-026-00899-y (PMC13640162; doi:10.1186/s40942-026-00899-y)
Supplement: Supplementary file 2 [file 40942_2026_899_MOESM2_ESM.docx]

**Supplementary File 2. All data extraction**

**ALL OUTCOME DATA**

**2A. BCVA outcomes**

**BCVA - naive / treatment-naive eyes**

| **Study** | **Subgroup** | **n** | **Format** | **Baseline** | **Final timepoint** | **Final value** | **Change** | **p-value** |
| --- | --- | --- | --- | --- | --- | --- | --- | --- |
| Danzig 2025 BALATON (linked Tadayoni 2024 wk 24) | Faricimab Q4W (naive BRVO) | 276 | Adj mean letters | 57.5 +/- 13.0 | Wk 24 | - | +16.9 letters (95.03% CI 15.7-18.1) | NI met vs afli |
| Danzig 2025 BALATON (linked Tadayoni 2024 wk 24) | Aflibercept Q4W (naive BRVO) | 277 | Adj mean letters | 57.6 +/- 12.2 | Wk 24 | - | +17.5 letters (95.03% CI 16.3-18.6) | - |
| Danzig 2025 BALATON | Faricimab Q4W (extended single-arm) | 276 | Adj mean letters | - | Wk 64-72 avg | - | +18.1 letters (95.03% CI 16.9-19.4) | - |
| Danzig 2025 BALATON | Aflibercept-to-fari (extended single-arm) | 277 | Adj mean letters | - | Wk 64-72 avg | - | +18.8 letters (95.03% CI 17.5-20.0) | - |
| Danzig 2025 COMINO (linked Tadayoni 2024 wk 24) | Faricimab Q4W (naive CRVO/HRVO) | 366 | Adj mean letters | 50.3 +/- 16.3 | Wk 24 | - | +16.9 letters (95.03% CI 15.4-18.3) | NI met vs afli |
| Danzig 2025 COMINO (linked Tadayoni 2024 wk 24) | Aflibercept Q4W (naive CRVO/HRVO) | 363 | Adj mean letters | 50.7 +/- 16.3 | Wk 24 | - | +17.3 letters (95.03% CI 15.9-18.8) | - |
| Danzig 2025 COMINO | Faricimab Q4W (extended single-arm) | 366 | Adj mean letters | - | Wk 64-72 avg | - | +16.9 letters (95.03% CI 15.2-18.6) | - |
| Danzig 2025 COMINO | Aflibercept-to-fari (extended single-arm) | 363 | Adj mean letters | - | Wk 64-72 avg | - | +17.1 letters (95.03% CI 15.4-18.8) | - |
| Wu 2026 | Naive CRVO | 29 | Mean logMAR / letters | logMAR 1.31 (Snellen 20/407) | Last visit (mean 26.2 wk) | logMAR 0.83 (20/136) | +22.6 letters (95% CI 9.3-36.0) | 0.00369 |
| Inokuchi 2025 | Naive mixed BRVO/CRVO | 5 | Median logMAR | 0.40 (IQR 0.30-0.40) | 3 months | 0.15 (IQR 0.00-0.40) | dlogMAR median 0.30; mean -0.25 | 0.068 (NS) |
| Hirakata 2025 | Naive BRVO plus CRVO | 17 | Mean logMAR +/- SD | 0.30 +/- 0.37 | Final (mean 3.7 mo) | 0.11 +/- 0.20 | - | 0.01 |
| Hikichi 2026 | Faricimab arm (naive BRVO) | 25 | Mean logMAR +/- SD | 0.40 +/- 0.30 | 6 months | ~0.09 (Figure 1; SD not separately tabulated) | - | NS between groups |
| Hikichi 2026 | Aflibercept arm (naive BRVO) | 29 | Mean logMAR +/- SD | 0.38 +/- 0.29 | 6 months | ~0.10 (Figure 1; SD not separately tabulated) | - | NS between groups |
| Hikichi 2026 | All eyes (combined) | 54 | Mean logMAR +/- SD | 0.39 +/- 0.29 | 1 mo: 0.16 +/- 0.26; 3 mo: 0.12 +/- 0.23; 6 mo: 0.09 +/- 0.23 | 0.09 +/- 0.23 | - | <0.001 vs baseline |

**BCVA - previously-treated / switch eyes**

| **Study** | **Subgroup** | **n** | **Format** | **Baseline** | **Final timepoint** | **Final value** | **Change** | **p-value** |
| --- | --- | --- | --- | --- | --- | --- | --- | --- |
| Hafner 2025 | Overall cohort | 19 | Median logMAR | 0.20 (IQR 0.50) | mo3: 0.00; mo9: 0.00 | 0.00 (IQR 0.20) | - | <0.01 mo0 vs mo9 |
| Hafner 2025 | Anti-VEGF pretreated subgroup | 14 | Median logMAR | 0.10 (IQR 0.25) | mo3: 0.00; mo9: 0.00 | 0.00 (IQR 0.20) | - | 0.02 mo0 vs mo9 |
| Wu 2026 | Pretreated CRVO | 21 | Mean letters | logMAR 1.02 (20/209) | Last visit (mean 21.8 wk) | logMAR 0.91 (20/162) | +9.8 letters (95% CI -0.2 to 19.8) | 0.5233 (NS) |
| Wu 2026 | Pretreated BRVO | 20 | Mean letters | logMAR 0.43 (20/54) | Last visit (mean 28.1 wk) | logMAR 0.34 (20/44) | +5.4 letters (95% CI -3.0 to 13.8) | 0.00862 |
| Inokuchi 2025 | Non-naive mixed | 18 | Median logMAR | 0.35 (IQR 0.17-0.52) | 3 months | 0.22 (IQR 0.08-0.38) | dlogMAR 0.12 | 0.0143 |
| Kilani 2026 | All RVO | 27 | Median logMAR | 0.2 (IQR 0.1-0.3); decimal 0.63 | Visit of 3rd injection (mean 11.7 wk) | 0.1 (IQR 0.1-0.2); decimal 0.8 | - | 0.022 |
| Kilani 2026 | CRVO subgroup | 12 | Median logMAR | 0.2 (IQR 0.1-0.3) | Visit of 3rd injection | 0.14 (IQR 0.1-0.4) | - | 0.1 (NS) |
| Kilani 2026 | BRVO subgroup | 15 | Median logMAR | 0.2 (IQR 0.1-0.3) | Visit of 3rd injection | 0.1 (IQR 0.2-0.7) [IQR appears anomalous] | - | 0.1 (NS) |
| Hirakata 2025 | Switch | 13 | Mean logMAR +/- SD | 0.45 +/- 0.45 | Final (mean 4.9 mo) | 0.35 +/- 0.37 | - | 0.19 (NS) |
| Hikichi 2025 | All switch | 42 | Mean logMAR +/- SE [SD] | 0.16 +/- 0.03 (SE) [SD 0.194] | 1 mo: 0.04 +/- 0.03 (SE); final (mean 8.3 mo): 0.02 +/- 0.02 (SE) [SD 0.130] | 0.02 +/- 0.02 (SE) | - | <0.01 |
| Hikichi 2025 | Best on prior therapy (pre-switch) | 42 | Mean logMAR +/- SE | 0.30 +/- 0.04 (SE) | - | - | - | <0.01 final vs best prior |
| Hikichi 2025 | Absence of fluid pre-switch | 42 | Mean logMAR +/- SE | 0.07 +/- 0.03 (SE) | - | - | - | 0.155 final vs absence-of-fluid pre-switch (NS) |
| Hikichi 2025 | CRVO subgroup | 17 | Mean logMAR +/- SE [SD] | 0.27 +/- 0.07 (SE) [SD 0.289] | 1 mo: 0.19 +/- 0.04 (SE); final: 0.15 +/- 0.03 (SE) [SD 0.124] | 0.15 +/- 0.03 (SE) | - | 1 mo p=0.051; final <0.01 |
| Hikichi 2025 | BRVO plus hemi subgroup | 25 | Mean logMAR +/- SE [SD] | 0.13 +/- 0.03 (SE) [SD 0.150] | 1 mo: 0.02 +/- 0.03 (SE); final: -0.02 +/- 0.02 (SE) [SD 0.100] | -0.02 +/- 0.02 (SE) | - | 1 mo <0.01; final <0.01 |
| Sun 2025 | Overall | 52 | Mean ETDRS letters +/- SD | 40.13 +/- 15.47 | Post-1st: 51.17 +/- 15.89; post-2nd: 50.40 +/- 16.59; post-3rd: 46.75 +/- 18.21 | 46.75 +/- 18.21 (post-3rd injection) | +11.04 (post-1st); +10.27 (post-2nd); +10.13 (post-3rd) | <0.001; <0.001; 0.043 |
| Sun 2025 | BRVO-ME | 18 | Mean letter change [derived SD] | - | Final | - | +7.20 +/- 7.53 (95% CI -2.15 to 16.55) [derived SD 20.23] | 0.367 vs CRVO |
| Sun 2025 | CRVO-ME | 34 | Mean letter change [derived SD] | - | Final | - | +11.05 +/- 8.49 (95% CI 6.96-15.14) [derived SD 12.16] | - |
| Sun 2025 | Ischaemic | 32 | Mean letter change | - | Final | - | +9.50 (95% CI 4.12-14.88) | 0.6112 vs non-ischaemic |
| Sun 2025 | Non-ischaemic | 20 | Mean letter change | - | Final | - | +11.30 (95% CI 6.32-16.28) | - |
| Wolfrum 2026 | Overall | 21 | Mean logMAR +/- SD | 0.50 +/- 0.52 | Post-1st: 0.40 +/- 0.32; post-2nd: 0.39 +/- 0.34; FU (~12 wk): 0.40 +/- 0.31 | 0.40 +/- 0.31 | - | 0.257 (NS) |
| Wolfrum 2026 | Full responder subgroup | 5 | Mean logMAR +/- SD | 1.0 +/- 0.8 | FU | 0.6 +/- 0.5 | - | Baseline diff p=0.041 |
| Wolfrum 2026 | Reduced responder subgroup | 16 | Mean logMAR +/- SD | 0.33 +/- 0.22 | FU | 0.34 +/- 0.23 | - | - |

**BCVA - proportion gaining/losing letters**

| **Study** | **Subgroup** | **n** | **Threshold** | **Proportion** |
| --- | --- | --- | --- | --- |
| Danzig 2025 BALATON (Tadayoni wk 24) | Faricimab Q4W | 276 | Gained >=15 letters at wk 24 | 56.1% |
| Danzig 2025 BALATON (Tadayoni wk 24) | Aflibercept Q4W | 277 | Gained >=15 letters at wk 24 | 60.4% |
| Danzig 2025 BALATON (Tadayoni wk 24) | Faricimab Q4W | 276 | Avoided loss >=15 letters at wk 24 | 99.6% |
| Danzig 2025 BALATON (Tadayoni wk 24) | Aflibercept Q4W | 277 | Avoided loss >=15 letters at wk 24 | 98.6% |
| Danzig 2025 BALATON (extended) | Prior faricimab Q4W | 276 | Gained >=15 letters wk 64-72 avg | 61.5% |
| Danzig 2025 BALATON (extended) | Prior aflibercept Q4W | 277 | Gained >=15 letters wk 64-72 avg | 65.8% |
| Danzig 2025 BALATON (extended) | Prior faricimab Q4W | 276 | Avoided loss >=15 letters wk 64-72 avg | 98.9% |
| Danzig 2025 BALATON (extended) | Prior aflibercept Q4W | 277 | Avoided loss >=15 letters wk 64-72 avg | 98.2% |
| Danzig 2025 COMINO (Tadayoni wk 24) | Faricimab Q4W | 366 | Gained >=15 letters at wk 24 | 56.6% |
| Danzig 2025 COMINO (Tadayoni wk 24) | Aflibercept Q4W | 363 | Gained >=15 letters at wk 24 | 58.1% |
| Danzig 2025 COMINO (Tadayoni wk 24) | Faricimab Q4W | 366 | Avoided loss >=15 letters at wk 24 | 96.2% |
| Danzig 2025 COMINO (Tadayoni wk 24) | Aflibercept Q4W | 363 | Avoided loss >=15 letters at wk 24 | 96.7% |
| Danzig 2025 COMINO (extended) | Prior faricimab Q4W | 366 | Gained >=15 letters wk 64-72 avg | 57.6% |
| Danzig 2025 COMINO (extended) | Prior aflibercept Q4W | 363 | Gained >=15 letters wk 64-72 avg | 59.5% |
| Danzig 2025 COMINO (extended) | Prior faricimab Q4W | 366 | Avoided loss >=15 letters wk 64-72 avg | 93.7% |
| Danzig 2025 COMINO (extended) | Prior aflibercept Q4W | 363 | Avoided loss >=15 letters wk 64-72 avg | 95.6% |
| Sun 2025 | Overall | 52 | Gained >=5 letters | 55.77% (29/52) |
| Sun 2025 | Overall | 52 | Gained >=10 letters | 28.85% (15/52) |
| Sun 2025 | Overall | 52 | Any improvement | 88.46% (46/52) |
| Inokuchi 2025 | Overall | 23 | Improved >=0.1 logMAR | 60.9% (14/23) |
| Inokuchi 2025 | Overall | 23 | Improved >=0.2 logMAR | 26.1% (6/23) |

**2B. CST/CMT outcomes**

**CST/CMT - naive eyes**

| **Study** | **Subgroup** | **n** | **Format** | **Baseline (microm)** | **Final (microm)** | **Change** | **p-value** |
| --- | --- | --- | --- | --- | --- | --- | --- |
| Danzig 2025 BALATON (Tadayoni wk 24) | Faricimab Q4W | 276 | Adj mean | 558.3 +/- 177.0 | Wk 24 | -311.4 microm (95.03% CI -316.4 to -306.4) | - |
| Danzig 2025 BALATON (Tadayoni wk 24) | Aflibercept Q4W | 277 | Adj mean | 558.1 +/- 180.3 | Wk 24 | -304.4 microm (95.03% CI -309.3 to -299.4) | - |
| Danzig 2025 BALATON (extended) | Prior faricimab Q4W | 276 | Adj mean | - | Wk 64-72 avg | -310.9 microm (95.03% CI -315.6 to -306.3) | - |
| Danzig 2025 BALATON (extended) | Prior aflibercept Q4W | 277 | Adj mean | - | Wk 64-72 avg | -307.0 microm (95.03% CI -311.7 to -302.3) | - |
| Danzig 2025 COMINO (Tadayoni wk 24) | Faricimab Q4W | 366 | Adj mean | 702.2 +/- 244.0 | Wk 24 | -461.6 microm (95.03% CI -471.4 to -451.9) | - |
| Danzig 2025 COMINO (Tadayoni wk 24) | Aflibercept Q4W | 363 | Adj mean | 721.1 +/- 242.9 | Wk 24 | -448.8 microm (95.03% CI -458.6 to -439.0) | - |
| Danzig 2025 COMINO (extended) | Prior faricimab Q4W | 366 | Adj mean | - | Wk 64-72 avg | -465.9 microm (95.03% CI -472.5 to -459.3) | - |
| Danzig 2025 COMINO (extended) | Prior aflibercept Q4W | 363 | Adj mean | - | Wk 64-72 avg | -460.6 microm (95.03% CI -467.2 to -453.9) | - |
| Wu 2026 | Naive CRVO | 29 | Mean +/- 95% CI | 491 (410-572) | 287 (229-344) at last visit | - | 0.0001 |
| Inokuchi 2025 | Naive | 5 | Median (IQR) | 407 (301-695) | 188 (144-194) at 3 mo | mean d 232.8 microm | 0.0625 (NS) |
| Hirakata 2025 | Naive | 17 | Mean +/- SD | 442 +/- 117 | 1 mo after final IVF: 304 +/- 57; final: 313 +/- 78 | - | 0.0002 / <0.0001 |
| Hikichi 2026 | All eyes | 54 | Mean CFT +/- SD | 617 +/- 132 | 1 mo: 218 +/- 94; 3 mo: 237 +/- 98; 6 mo: 201 +/- 68 | - | <0.001 |
| Hikichi 2026 | Faricimab arm | 25 | Mean CFT +/- SD | 598 +/- 106 | 1 mo: 207 +/- 86; 3 mo: 201 +/- 63; 6 mo: 192 +/- 57 | - | <0.001 |
| Hikichi 2026 | Aflibercept arm | 29 | Mean CFT +/- SD | 635 +/- 161 | 1 mo: 227 +/- 102; 3 mo: 275 +/- 131; 6 mo: 223 +/- 80 | - | <0.001 |

**CST/CMT - switch eyes**

| **Study** | **Subgroup** | **n** | **Format** | **Baseline (microm)** | **Final (microm)** | **Change** | **p-value** |
| --- | --- | --- | --- | --- | --- | --- | --- |
| Hafner 2025 | Overall | 19 | Median CST (IQR) | 325 (160) | mo3: 285 (40); mo9: 285 (48) | - | <0.01 mo0 vs mo9 |
| Hafner 2025 | Anti-VEGF pretreated | 14 | Median CST (IQR) | 310 (116) | mo3: 282 (31); mo9: 282 (47) | - | <0.01 mo0 vs mo9 |
| Wu 2026 | Pretreated CRVO | 21 | Mean +/- 95% CI | 498 (401-595) | 347 (266-428) at last visit | - | (CST p not stated for this subgroup) |
| Wu 2026 | Pretreated BRVO | 20 | Mean +/- 95% CI | 351 (281-421) | 274 (236-312) at last visit | - | 0.01753 |
| Inokuchi 2025 | Non-naive | 18 | Median (IQR) | 343 (214.8-426.5) | 210 (165.8-289.5) at 3 mo | mean d 43.5 microm | 0.0016 |
| Kilani 2026 | Overall | 27 | Median (IQR) | 291 (281-325) | 268 (253-289) at visit of 3rd injection | - | <0.001 |
| Kilani 2026 | CRVO | 12 | Median (IQR) | 312 (283-372) | 263 (253-276) | - | 0.002 |
| Kilani 2026 | BRVO | 15 | Median (IQR) | 289 (280-302) | 270 (253-290) | - | 0.08 (NS) |
| Hirakata 2025 | Switch | 13 | Mean +/- SD | 436 +/- 170 | 1 mo after final IVF: 285 +/- 76; final: 271 +/- 71 | - | <0.05 / 0.0002 |
| Hikichi 2025 | All switch | 42 | Mean CFT +/- SE [SD] | 356 +/- 23 (SE) [SD 149.0] | 1 mo: 214 +/- 3 (SE) [SD 19.4]; final: 205 +/- 4 (SE) [SD 25.9] | - | <0.01 / <0.01 |
| Hikichi 2025 | Absence of fluid pre-switch | 42 | Mean CFT +/- SE | 239 +/- 12 (SE) | - | - | 0.248 final vs absence-of-fluid pre-switch (NS) |
| Hikichi 2025 | CRVO | 17 | Mean CFT +/- SE [SD] | 388 +/- 22 (SE) [SD 90.7] | 1 mo: 249 +/- 8 (SE); final: 221 +/- 8 (SE) [SD 33.0] | - | 0.02 / <0.01 |
| Hikichi 2025 | BRVO plus hemi | 25 | Mean CFT +/- SE [SD] | 347 +/- 13 (SE) [SD 65.0] | 1 mo: 198 +/- 5 (SE); final: 199 +/- 4 (SE) [SD 25.0] | - | <0.01 / <0.01 |
| Sun 2025 | Overall | 52 | Mean CMT +/- SD | 547.40 +/- 160.9 | Post-1st: 251.0 +/- 91.93; post-2nd: 276.4 +/- 90.49; post-3rd: 216.4 +/- 105.3 | d -323.4 (post-1st); d -298.0 (post-2nd); d -358.0 (post-3rd) | <0.001; <0.001; 0.003 |
| Sun 2025 | BRVO-ME | 18 | Mean CMT change [derived SD] | - | Final | -316.8 +/- 155.4 (95% CI -509.8 to -123.8) [derived SD 417.5; flag for sense-check] | 0.806 vs CRVO |
| Sun 2025 | CRVO-ME | 34 | Mean CMT change [derived SD] | - | Final | -325.3 +/- 207 (95% CI -425.1 to -225.5) [derived SD 296.8] | - |
| Sun 2025 | Ischaemic | 32 | Mean CMT change | - | Final | -388.3 (95% CI -503.3 to -273.3) | 0.053 vs non-ischaemic |
| Sun 2025 | Non-ischaemic | 20 | Mean CMT change | - | Final | -232.9 (95% CI -342.5 to -123.3) | - |
| Wolfrum 2026 | Overall | 21 | Mean CRT +/- SD | 350.2 +/- 124.5 | FU (~12 wk): 302.6 +/- 74.6 | d -47.6 microm (median d -14, IQR -63 to -2) | 0.037 |
| Wolfrum 2026 | Full responders | 5 | Mean CRT +/- SD | 468.0 +/- 162.9 | FU | 307.2 +/- 82.5 | Baseline diff p=0.026 |
| Wolfrum 2026 | Reduced responders | 16 | Mean CRT +/- SD | 313.4 +/- 86.7 | FU | 301.2 +/- 74.8 | - |
| Wolfrum 2026 | Overall | 21 | Mean CRV +/- SD (mm^3) | 2.53 +/- 0.46 | FU | 2.34 +/- 0.29 | 0.011 |

**2C. Anatomical resolution / fluid status**

| **Study** | **Subgroup** | **n** | **Outcome** | **Result** |
| --- | --- | --- | --- | --- |
| Danzig 2025 BALATON (Tadayoni wk 24) | Faricimab Q4W | 229 evaluable FA | Absence of FA leakage at wk 24 | 33.6% (nominal p=0.0023 vs afli) |
| Danzig 2025 BALATON (Tadayoni wk 24) | Aflibercept Q4W | 224 evaluable FA | Absence of FA leakage at wk 24 | 21.0% |
| Danzig 2025 COMINO (Tadayoni wk 24) | Faricimab Q4W | 311 evaluable FA | Absence of FA leakage at wk 24 | 44.4% (nominal p=0.0002 vs afli) |
| Danzig 2025 COMINO (Tadayoni wk 24) | Aflibercept Q4W | 297 evaluable FA | Absence of FA leakage at wk 24 | 30.0% |
| Hafner 2025 | Overall | 19 | IRF proportion baseline / mo3 / mo9 | 100% / 26% / 26% (p<0.01 mo0 vs mo9) |
| Hafner 2025 | Anti-VEGF pretreated | 14 | IRF proportion baseline / mo3 / mo9 | 100% / 14% / 14% (p<0.01) |
| Kilani 2026 | Overall | 27 | IRF: baseline to final | 100% to 52% (p<0.001) |
| Kilani 2026 | Overall | 27 | Dry macula at final visit | 13/27 (48.1%) |
| Kilani 2026 | CRVO | 12 | IRF: baseline to final | 100% to 41.7% (p=0.016) |
| Kilani 2026 | BRVO | 15 | IRF: baseline to final | 100% to 60% (p=0.031) |
| Hikichi 2025 | All switch | 42 | IRF status: 1 mo / 3 mo / final | 14.3% / 50% / 35.7% (1 with SRF at final) |
| Hikichi 2026 | All eyes | 54 | IRF and SRF status at baseline | IRF in 54/54; SRF in 38/54 (70.4%); CFT mean 201 microm at 6 mo |
| Inokuchi 2025 | Overall | 23 | Complete fluid resolution by 3 mo | 12/23 (52.2%) |
| Inokuchi 2025 | Naive | 5 | Complete fluid resolution | 4/5 (80%) |
| Inokuchi 2025 | Non-naive | 18 | Complete fluid resolution | 8/18 (44.4%) |
| Sun 2025 | Overall | 52 | Complete fluid resolution at final | 41/52 (78.85%) of those with baseline fluid |
| Sun 2025 | Overall | 52 | >=20% CMT reduction at final | 52/52 (100%) |
| Hirakata 2025 | Naive | 17 | No fluid on OCT 1 mo after first IVF | 12/17 (71%) |
| Hirakata 2025 | Naive | 17 | CMT <300 microm 1 mo after final IVF | 12/17 (70.6%) |
| Hirakata 2025 | Switch | 13 | No fluid on OCT 1 mo after first IVF | 6/13 (46%) |
| Hirakata 2025 | Switch | 13 | CMT <250 microm 1 mo after final IVF | 6/13 (46.2%) |
| Wolfrum 2026 | Overall | 21 | Complete retinal dryness at FU | 7/21 (33.3%) |

**2D. Treatment intervals and injection burden**

| **Study** | **Subgroup** | **n** | **Outcome** | **Result** |
| --- | --- | --- | --- | --- |
| Danzig 2025 BALATON | Prior faricimab Q4W | 276 | Mean injections wk 24-72 | Median 4 (mean 4.8 +/- 2.6) |
| Danzig 2025 BALATON | Prior aflibercept Q4W | 277 | Mean injections wk 24-72 | Median 4 (mean 4.9 +/- 2.8) |
| Danzig 2025 BALATON | Prior faricimab Q4W | 248 | At wk 68: Q4W / Q8W / Q12W / Q16W | 23% / 13% / 12% / 52% (>=Q12W = 64%) |
| Danzig 2025 BALATON | Prior aflibercept Q4W | 244 | At wk 68: Q4W / Q8W / Q12W / Q16W | 25% / 18% / 9% / 48% (>=Q12W = 57%) |
| Danzig 2025 BALATON | Combined | - | Achieved >=Q12W at any point wk 24-68 | 81.5% |
| Danzig 2025 BALATON | Combined | - | Maintained >=Q12W after first cycle | 72.1% |
| Danzig 2025 COMINO | Prior faricimab Q4W | 366 | Mean injections wk 24-72 | Median 5 (mean 5.6 +/- 3.0) |
| Danzig 2025 COMINO | Prior aflibercept Q4W | 363 | Mean injections wk 24-72 | Median 4 (mean 5.5 +/- 2.9) |
| Danzig 2025 COMINO | Prior faricimab Q4W | 330 | At wk 68: Q4W / Q8W / Q12W / Q16W | 35% / 20% / 8% / 37% (>=Q12W = 45%) |
| Danzig 2025 COMINO | Prior aflibercept Q4W | 315 | At wk 68: Q4W / Q8W / Q12W / Q16W | 32% / 17% / 11% / 39% (>=Q12W = 50%) |
| Danzig 2025 COMINO | Combined | - | Achieved >=Q12W at any point wk 24-68 | 74.0% |
| Danzig 2025 COMINO | Combined | - | Maintained >=Q12W after first cycle | 61.6% |
| Hafner 2025 | Anti-VEGF pretreated | 14 | Median interval baseline to mo9 (days) | 45.5 to 56.5 (p=0.01; Cliff d=0.86); approx 6.5 to 8.07 weeks |
| Hafner 2025 | Anti-VEGF pretreated | 14 | At mo9: distribution | 4-6wk: 7.1%; 6-8wk: 35.7%; 8-10wk: 35.7%; 10-12wk: 7.1%; >12wk: 14.3%; >=Q12W = 21.4%; >=Q16W = 0% |
| Wu 2026 | Naive CRVO | 29 | Mean interval at last visit (weeks) | 8.8 (95% CI 5.6-11.9) |
| Wu 2026 | Pretreated CRVO | 21 | Mean interval at last visit (weeks) | 11.0 (95% CI 7.5-14.5) |
| Wu 2026 | Pretreated BRVO | 20 | Mean interval at last visit (weeks) | 11.1 (95% CI 6.6-15.6) |
| Wu 2026 | Naive CRVO | 29 | Mean injections | 3.7 (95% CI 2.6-4.8) |
| Wu 2026 | Pretreated CRVO | 21 | Mean injections | 3.4 |
| Wu 2026 | Pretreated BRVO | 20 | Mean injections | 3.5 |
| Inokuchi 2025 | Overall | 23 | Median injections over 3 mo | 1 (IQR 1-1); mean 1.4 (range 1-3); 78.3% had only 1 |
| Kilani 2026 | Overall | 27 | Mean interval baseline to final (weeks) | 4.6 +/- 0.8 to 7.3 +/- 1.6 (p<0.001) |
| Kilani 2026 | Overall | 27 | Median intended interval extension for next injection | 2 weeks (IQR 2-4); 88.9% had extension |
| Kilani 2026 | CRVO | 12 | Mean interval baseline to final | 4.8 +/- 0.9 to 7.3 +/- 1.6 (p=0.02); 100% had extension |
| Kilani 2026 | BRVO | 15 | Mean interval baseline to final | 4.4 +/- 0.7 to 6.7 +/- 0.7 (p=0.02); 80% had extension |
| Hirakata 2025 | Naive | 17 | Mean injections / FU | 1.4 +/- 0.7 / 3.7 +/- 2.7 mo |
| Hirakata 2025 | Switch | 13 | Mean injections / FU | 2.4 +/- 2.1 / 4.9 +/- 2.9 mo |
| Hikichi 2025 | All switch | 42 | Mean interval baseline to final (weeks) | 12.3 +/- 0.4 (SE) [SD 2.59] to 16.2 +/- 0.5 (SE) [SD 3.24] (p<0.01; mean diff 3.9 weeks); ranges 10-24 to 8-20 |
| Hikichi 2026 | Faricimab arm | 25 | Mean injections over 6 mo | 1.96 +/- 0.68 |
| Hikichi 2026 | Aflibercept arm | 29 | Mean injections over 6 mo | 2.45 +/- 0.87 (p=0.024 vs faricimab) |
| Hikichi 2026 | Faricimab arm | 25 | Retreatment-free at 1/2/3/6 mo | 88% / 80% / 60% / 24% |
| Hikichi 2026 | Aflibercept arm | 29 | Retreatment-free at 1/2/3/6 mo | 79.3% / 62.1% / 27.6% / 13.8% |
| Hikichi 2026 | Faricimab arm | 25 | No additional injections after first | 6/25 (24%) |
| Hikichi 2026 | Aflibercept arm | 29 | No additional injections after first | 4/29 (13.8%) |
| Sun 2025 | Overall | 52 | Mean injections / FU | 2.97 +/- 0.74 / 25.65 +/- 11.87 weeks |
| Sun 2025 | Overall | 52 | Mean treatment interval at final | 14.67 +/- 6.42 weeks |
| Sun 2025 | Overall | 52 | Proportion at interval immediately preceding final injection: >=8wk / >=12wk / >=16wk | 73.08% / 42.31% / 30.77% |
| Sun 2025 | BRVO-ME | 18 | Mean injections | 2.44 +/- 0.73 |
| Sun 2025 | CRVO-ME | 34 | Mean injections | 3.13 +/- 0.68 |
| Sun 2025 | BRVO subgroup | 18 | Mean interval at final (weeks) | 12.8 +/- 3.35 |
| Sun 2025 | CRVO subgroup | 34 | Mean interval at final (weeks) | 11.79 +/- 3.39 |
| Sun 2025 | Ischaemic | 32 | Mean interval at final (weeks) | 11.56 +/- 4.22 |
| Sun 2025 | Non-ischaemic | 20 | Mean interval at final (weeks) | 12.86 +/- 2.80 |
| Wolfrum 2026 | Overall | 21 | Mean injections | 3 (fixed by protocol; no maintenance phase analysed) |

**2E. Safety**

All 10 studies report no retinal vasculitis. Specific events below.

| **Study** | **n** | **IOI / uveitis** | **RAO / vascular** | **IOP** | **Cataract / other** |
| --- | --- | --- | --- | --- | --- |
| Danzig BALATON wk 0-24 (Tadayoni 2024, faricimab Q4W) | 276 | 1 (0.4%) IOI event (verbatim "vitreal cells", reported as non-inflammatory) | 0 RAO; 0 embolism | Not separately tabulated | RVO worsening 1 (0.4%); APTC events 3 (1.1%) |
| Danzig BALATON wk 0-24 (Tadayoni 2024, aflibercept Q4W) | 274 | 0 IOI | 0 | - | RVO worsening 3 (1.1%); APTC events 4 (1.5%) |
| Danzig COMINO wk 0-24 (Tadayoni 2024, faricimab Q4W) | 365 | 8 (2.2%) IOI events; 2 serious uveitis (1 with sustained 24-letter loss after 3 inj plus worsening CME, 1 after 4 inj with preserved BCVA) | RAO 3 (0.8%); 0 embolism | - | RVO worsening 1 (0.3%); APTC events 4 (1.1%) |
| Danzig COMINO wk 0-24 (Tadayoni 2024, aflibercept Q4W) | 361 | 4 (1.1%) IOI events; 1 serious noninfectious endophthalmitis (after 1 inj, recovered) | RAO 1 (0.3%); 1 embolism (0.3%) | - | RVO worsening 5 (1.4%); APTC events 5 (1.4%); 1 endophthalmitis (after 3 inj, 45-letter loss, not recovered) |
| Danzig BALATON wk 24-72 (faricimab Q4W) | 270 | 2 (0.7%); all nonserious, mild, resolved | 0 RAO; 1 retinal artery embolism (Hollenhorst) | Not separately tabulated | RVO worsening 9 (3.3%); APTC events 2 (0.7%) |
| Danzig BALATON wk 24-72 (aflibercept Q4W to fari) | 267 | 3 (1.1%); all nonserious, mild | 0 RAO; 0 embolism | - | RVO worsening 9 (3.4%); APTC events 8 (3.0%) |
| Danzig COMINO wk 24-72 (faricimab Q4W) | 359 | 10 (2.8%); 3 serious (uveitis, vitritis, iridocyclitis); 1 vitritis with sustained >=15-letter loss | RAO 2 (0.6%); 1 embolism | - | RVO worsening 11 (3.1%); APTC events 8 (2.2%); 1 noninfectious endophthalmitis |
| Danzig COMINO wk 24-72 (aflibercept Q4W to fari) | 342 | 5 (1.5%); 1 iridocyclitis with transient 29-letter loss (recovered) | RAO 2 (0.6%); 0 embolism | - | RVO worsening 8 (2.3%); APTC events 6 (1.8%) |
| Hafner 2025 | 19 | 0 IOI/uveitis | 0 | Median IOP unchanged 16 mmHg throughout (p=0.81) | No cataract surgery; no AEs |
| Wu 2026 (RVO subset) | 70 | 1 anterior uveitis (in pretreated CRVO; topical steroids; switched back to aflibercept) | 0 | Not reported | 0 endophthalmitis |
| Inokuchi 2025 | 23 | 0 | 0 | 0 IOP elevation | 0 RD, 0 vitreous opacity, 0 systemic AEs |
| Kilani 2026 | 27 | 0 | 0 | Not reported | 0 safety AEs |
| Hirakata 2025 | 30 | 0 | 0 | Stable in both groups | 0 RD, 0 systemic AEs |
| Hikichi 2026 | 54 | 0 | 0 | No prolonged IOP elevation | 0 cataract surgery, 0 endophthalmitis |
| Hikichi 2025 | 42 | 0 | 0 | Not reported | 0 ocular or systemic AEs |
| Sun 2025 | 52 | 0 | 0 | 0 IOP elevation | 0 endophthalmitis, 0 vitreous haemorrhage, 0 retinal tears, 0 cataract exacerbation, 0 serious systemic AEs |
| Wolfrum 2026 | 21 | Not structured as outcome | - | - | - |
