# Supplementary material for: Effectiveness and safety of intravitreal faricimab for macular oedema secondary to retinal vein occlusion: a systematic review and meta-analysis
Source: Int J Retina Vitreous. 2026 Jul 9;12:134. doi: 10.1186/s40942-026-00899-y (PMC13640162; doi:10.1186/s40942-026-00899-y)
Supplement: Supplementary file 3 [file 40942_2026_899_MOESM3_ESM.docx]

# **Supplementary File 3: Database Search Strategies**

Effectiveness and safety of intravitreal faricimab for macular oedema secondary to retinal vein occlusion: a systematic review and meta-analysis

## **Overview**

Three electronic bibliographic databases were systematically searched on 1 May 2026: PubMed, EMBASE, and the Cochrane Central Register of Controlled Trials (CENTRAL). The same conceptual search strategy was applied across all three databases, combining free-text terms for the intervention (faricimab and synonyms) with free-text terms for the indication (retinal vein occlusion and macular oedema). Controlled vocabulary terms (MeSH headings, Emtree terms) were not separately incorporated into the search; the search strategy relied on free-text terms with synonyms and known alternative names for faricimab including its development codes (RG-7716, RO-6867461) and pharmacological descriptors.

No language, date, or study design restrictions were applied at the search stage. Eligibility criteria were assessed during title/abstract screening and full-text review in accordance with the prespecified inclusion and exclusion criteria documented in the registered protocol (PROSPERO CRD420261384239) and reported in the manuscript Methods section.

## **Summary of search results**

| **Database** | **Platform/interface** | **Date searched** | **Records returned** |
| --- | --- | --- | --- |
| PubMed | PubMed | 1 May 2026 | 234 |
| EMBASE | EMBASE | 1 May 2026 | 575 |
| Cochrane CENTRAL | Cochrane Library | 1 May 2026 | 78 |
| **Total (before deduplication)** |  |  | **887** |
| Duplicate records removed |  |  | 293 |
| **Total (after deduplication)** |  |  | **594** |

## **PubMed**

**Platform/interface:** PubMed

**Date search run:** 1 May 2026

**Date range:** From inception to 1 May 2026

**Filters or limits applied:** None at the search stage. Language restrictions, date restrictions, and study design restrictions were not applied at the search level. Eligibility was assessed at the screening and full-text review stages in accordance with the prespecified inclusion and exclusion criteria.

**Records returned:** 234

**Search string:**

*Line 1 (faricimab concept):*

(faricimab OR Vabysmo OR "RG-7716" OR "RG7716" OR "RO-6867461" OR "RO6867461" OR "anti-VEGF/Ang2" OR "bispecific antibody")

*Line 2 (retinal vein occlusion / macular oedema concept):*

(retinal vein occlusion OR RVO OR BRVO OR CRVO OR HRVO OR "branch retinal vein occlusion" OR "central retinal vein occlusion" OR "hemiretinal vein occlusion" OR "hemi-retinal vein occlusion" OR "macular edema" OR "macular oedema" OR "cystoid macular edema")

*Line 3 (final combined search):*

Line 1 AND Line 2

## **EMBASE**

**Platform/interface:** EMBASE

**Date search run:** 1 May 2026

**Date range:** From inception to 1 May 2026

**Filters or limits applied:** None at the search stage. Language restrictions, date restrictions, and study design restrictions were not applied at the search level. Eligibility was assessed at the screening and full-text review stages in accordance with the prespecified inclusion and exclusion criteria.

**Records returned:** 575

**Search string:**

*Line 1 (faricimab concept):*

(faricimab OR Vabysmo OR "RG-7716" OR "RG7716" OR "RO-6867461" OR "RO6867461" OR "anti-VEGF/Ang2" OR "bispecific antibody")

*Line 2 (retinal vein occlusion / macular oedema concept):*

(retinal vein occlusion OR RVO OR BRVO OR CRVO OR HRVO OR "branch retinal vein occlusion" OR "central retinal vein occlusion" OR "hemiretinal vein occlusion" OR "hemi-retinal vein occlusion" OR "macular edema" OR "macular oedema" OR "cystoid macular edema")

*Line 3 (final combined search):*

Line 1 AND Line 2

## **Cochrane Central Register of Controlled Trials**

**Platform/interface:** Cochrane Library

**Date search run:** 1 May 2026

**Date range:** From inception to 1 May 2026

**Filters or limits applied:** None at the search stage. Language restrictions, date restrictions, and study design restrictions were not applied at the search level. Eligibility was assessed at the screening and full-text review stages in accordance with the prespecified inclusion and exclusion criteria.

**Records returned:** 78

**Search string:**

*Line 1 (faricimab concept):*

(faricimab OR Vabysmo OR "RG-7716" OR "RG7716" OR "RO-6867461" OR "RO6867461" OR "anti-VEGF/Ang2" OR "bispecific antibody")

*Line 2 (retinal vein occlusion / macular oedema concept):*

(retinal vein occlusion OR RVO OR BRVO OR CRVO OR HRVO OR "branch retinal vein occlusion" OR "central retinal vein occlusion" OR "hemiretinal vein occlusion" OR "hemi-retinal vein occlusion" OR "macular edema" OR "macular oedema" OR "cystoid macular edema")

*Line 3 (final combined search):*

Line 1 AND Line 2

## **Methodological notes on the search strategy**

**Free-text search approach:**

The search strategy used free-text terms across all three databases rather than separately incorporating database-specific controlled vocabulary headings (MeSH for PubMed, Emtree for EMBASE). This approach was adopted because faricimab is a recently approved compound (United States 2022 for neovascular AMD; 2023 for retinal vein occlusion), and at the time of search execution (1 May 2026) the controlled vocabulary indexing of faricimab and its development codes was not yet uniformly established across all databases. The free-text search captured faricimab by its generic name, brand name (Vabysmo), and known development codes (RG-7716, RO-6867461) to ensure comprehensive identification of relevant literature regardless of indexing status.

**Hand-searching:**

Reference lists of included studies and relevant systematic reviews were hand-searched to identify additional eligible records not retrieved through the database searches. No additional studies were identified through hand-searching that met the eligibility criteria beyond those identified in the primary database searches.
